# Nanoparticles for Targeted Drug Delivery to Cancer Stem Cells: A Review of Recent Advances

**DOI:** 10.3390/nano11071755

**Published:** 2021-07-05

**Authors:** Yavuz Nuri Ertas, Keyvan Abedi Dorcheh, Ali Akbari, Esmaiel Jabbari

**Affiliations:** 1 Department of Biomedical Engineering, Erciyes University, Kayseri 38039, Turkey; yavuzertas@erciyes.edu.tr; 2 ERNAM—Nanotechnology Research and Application Center, Erciyes University, Kayseri 38039, Turkey; 3 Department of Biomedical Engineering, Faculty of Chemical Engineering, Tarbiat Modares University, Tehran 14115, Iran; keyvan.abedi@modares.ac.ir; 4 Solid Tumor Research Center, Research Institute for Cellular and Molecular Medicine, Urmia University of Medical Sciences, Urmia 57147, Iran; akbari.a@umsu.ac.ir; 5 Biomaterials and Tissue Engineering Laboratory, Department of Chemical Engineering, University of South Carolina, Columbia, SC 29208, USA

**Keywords:** targeted cancer therapy, cancer stem cells, nanoparticles, polymers, nanocarriers, self-assembling proteins, nanovesicles, dual-targeted drug delivery

## Abstract

Cancer stem cells (CSCs) are a subpopulation of cells that can initiate, self-renew, and sustain tumor growth. CSCs are responsible for tumor metastasis, recurrence, and drug resistance in cancer therapy. CSCs reside within a niche maintained by multiple unique factors in the microenvironment. These factors include hypoxia, excessive levels of angiogenesis, a change of mitochondrial activity from aerobic aspiration to aerobic glycolysis, an upregulated expression of CSC biomarkers and stem cell signaling, and an elevated synthesis of the cytochromes P450 family of enzymes responsible for drug clearance. Antibodies and ligands targeting the unique factors that maintain the niche are utilized for the delivery of anticancer therapeutics to CSCs. In this regard, nanomaterials, specifically nanoparticles (NPs), are extremely useful as carriers for the delivery of anticancer agents to CSCs. This review covers the biology of CSCs and advances in the design and synthesis of NPs as a carrier in targeting cancer drugs to the CSC subpopulation of cancer cells. This review includes the development of synthetic and natural polymeric NPs, lipid NPs, inorganic NPs, self-assembling protein NPs, antibody-drug conjugates, and extracellular nanovesicles for CSC targeting.

## 1. Introduction

According to a report by the World Health Organization (WHO), cancer is recognized as the second leading cause of death in the world, with over 18 million cases and close to 10 million cancer-related mortalities in 2018 [1]. Due to the rapid pace of industrialization, it is anticipated that cancer mortality rates will nearly double by 2040 [2]. Conventional cancer therapies, such as surgical resection of tumor, radiotherapy, and chemotherapy, not only destroy tumor cells, but they also harm healthy cells in cancer patients, leading to many undesired side effects, such as a loss of appetite, anemia, internal bleeding, and fatigue [3]. Among the cells within a tumor, there is a small subpopulation, typically less than one percent, that are highly resistant to conventional therapies. These cells are called cancer stem cells (CSCs) or cancer-initiating cells (CICs). The existence of CSCs, with their unique properties and cellular markers, has been reported in a broad range of cancers, including breast [4], colon [5], lung [6], prostate [7], liver [8], melanoma [9], leukemia [10], head and neck [11], ovarian [12], pancreatic [13], and brain tumors [14]. CSCs provide a unique strategy to treat patients with highly resistant, metastatic, and malignant cancers. To this end, the multidisciplinary field of nanotechnology promises new approaches to cancer treatment by targeting therapeutics to CSCs, the most resistant cells in the tumor tissue, thus potentially eliminating the undesired effects of therapeutics [15]. Recent years have witnessed the development of various organic and inorganic nanocarriers, with different sizes and shapes, as promising tools for CSC targeted therapies [16]. This review aims to summarize new trends and developments in various nanomaterials, including organic and inorganic nanoparticles (NPs), for targeting CSCs.

## 2. Cancer Stem Cell Biology

Cancer is defined as a biological condition in which some cells in a tissue of a bodily organ undergo an uncontrolled division and growth [3]. In 1997, Bonnet and Dick realized that a small subpopulation of these abnormal cells have different properties from those of bulk tumor cells. After isolation, they demonstrated that this small population of leukemia-initiating cells have features similar to stem cells and announced the concept of cancer stem cells (CSCs) [17]. Later studies in various types of solid tumors revealed the existence of CSCs in almost all cancer types, from brain to colon and prostate. The majority of cells in bulk tumors are normal and non-tumorigenic and behave like background cells with no special privileges, compared to CSCs [18,19]. CSCs can be compared with normal stem cells in different tissues of the body. Normal stem cells, when activated, undergo an asymmetric cell division (ACD) to self-renew and give rise to a distinct population of progenitors. These progenitors then undergo a symmetric cell division (SCD) to clonally expand and replenish lost cells [20]. CSCs in some ways act like normal stem cells for the tumor tissue. Evidence shows that normal cancer cells exhibit plasticity and undergo dedifferentiation to a stem-like state, like the epithelial-to-mesenchymal transition (EMT). These dedifferentiated cells acquire properties of stemness and become more invasive and metastatic. A key characteristic of CSCs is their ability to evade the attack by immune cells, like natural killer (NK) and CD8-positive cytotoxic T cells, through the active recruitment of immune suppression cells, expression of immune suppressive factors, or induction of apoptosis in T lymphocytes [21]. Other important features of CSCs include:

- Self-renewal and DNA repair: this extraordinary property of CSCs causes tumor relapse and radiation-resistance in tumors [22].
- Differentiation into multiple cell types: the pluripotency of CSCs causes heterogeneity in solid tumors [23].
- Ionizing radiation: this feature makes CSCs resistant to radiotherapy.
- Infinite proliferative potential: unlimited cell division, which leads to rapid tumor growth.
- Dormancy state: CSCs enter dormancy to evade the attack by the immune system, awaiting new signals from the environment to re-enter the cell cycle [22].
- Changes in morphology or biological function, such as the over-expression of anti-apoptotic proteins that block the cell from entering the type I apoptosis cycle [1,22,24].
- Elevated expression of ATP-binding cassette (ABC) pumps and detoxifying enzymes to increase the drug’s efflux, which is considered to be an important mechanism for multi-drug resistance (MDR). Multi-drug resistance is either intrinsic and present before the start of treatment or acquired after exposure to treatment [25].

**Figure 1:**
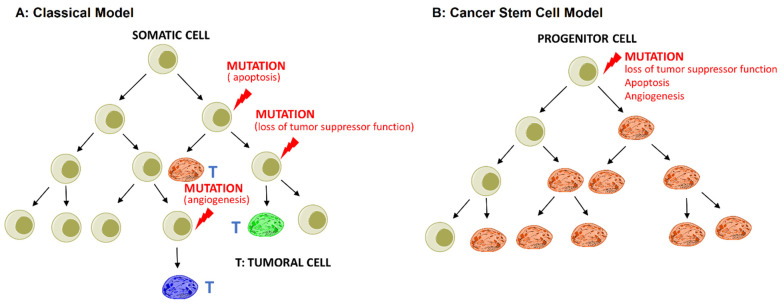
Two models of cancer development: (**A**) the stochastic or classical model; (**B**) the hierarchical or cancer stem cell model. Reprinted with permission from ref. [22], Copyright 2017 Elsevier.

While the exact mechanism of CSC initiation is unclear, there are two proposed theoretical models to explain their existence in tumor tissue:

1. The stochastic or classical model states that any somatic cell has the intrinsic ability to undergo mutation and transform into CSCs driven by genetic instability or environmental signals, as shown in Figure 1A;
2. The hierarchical or cancer stem cell model states that the initiating cancer cell self-renews in the process of cell division and forms a CSC and a normal cancer cell. The normal cancer cell divides and generates the cells in bulk tumors, as shown in Figure 1B [22,26].

### 2.1. Extracellular Matrix (ECM)

The ECM in normal body tissues is a collection of tightly regulated soluble and insoluble biomolecules with a defined composition, which is regulated by intracellular signaling pathways and expression levels. Conversely, the composition of the tumor ECM consisting of different collagen types and other components, as well as their connection with cells through ligand–receptor interactions, is abnormal. This abnormal ECM environment serves as the niche for the maintenance of CSCs [21].

### 2.2. CSC Niche

CSCs reside within a niche in the tumor tissue. The niche is an intrinsically dynamic system formed by the tumor microenvironment, with specific anatomical and functional features to maintain the CSCs [17]. A common feature of different tumors is hypoxia, which results from the abnormal growth of cancer cells and aberrant angiogenesis [25,27]. The nutrient deficiency created by aberrant cell growth instructs CSCs to activate the autophagy process, namely, type II programmed cell death, to restore the ATP energy level required for the metabolism of other cells [12]. As postulated by the “seed-soil” theory, cells with tumorigenic potential, depending on their microenvironment, express surface markers and differentiate into lineages that are different from normal cells [8].

### 2.3. Tumor Angiogenesis

Due to hypoxic conditions, tumor growth and metastasis require disproportionate levels of angiogenesis. As a result, CSCs express high levels of angiogenic factors, such as vascular endothelial growth factor (VEGF) and stromal derived factor-1 (SDF-1), to stimulate vascularization. This rapid vascularization results in the formation of disordered vessels with relatively large intracellular clefts between endothelial cells [6], which leads to an enhanced permeability and retention of nanomaterials, such as liposomes, self-assembled NPs, and drug-polymer conjugates, in the tumor tissue.

### 2.4. Mitochondrial Activity of CSCs

Another important characteristic of CSCs is their ability to change their metabolic activity and mitochondrial function to enhance drug resistance and cell survival. Mitochondria have been shown to play a key role in cell survival, as there is a close correlation between mitochondrial activity and cell pluripotency. The dynamic metabolic state of mitochondria in CSCs, namely, the shift from aerobic respiration to aerobic glycolysis, enables their survival under hypoxic conditions, as well as other metabolic stresses. The shift to aerobic glycolysis in the mitochondria of CSCs can be used for the recognition and targeting of cancer therapeutics [28]. Another important characteristic of CSCs is their unique surface markers and intracellular pathways, which can be used for drug targeting. These pathways enable CSCs to evade and survive radiation and chemotherapy and trigger cancer relapse [3].

### 2.5. Surface Biomarkers

The biomarkers for CSCs vary depending on the tissue of origin, but the most well-known CSC markers are CD44, CD90, CD133, and aldehyde dehydrogenase (ALDH), with each marker playing a role in CSC maintenance. CD44, a common marker among many cancer types, is a transmembrane hyaluronic acid receptor involved in cell adhesion, migration, metastasis, and drug resistance [3,18,26]. CD90 is a glycosyl phosphatidylinositol-anchored membrane glycoprotein, which is mainly expressed in leukocytes. CD90-positive cells possess tumorigenicity and metastatic potential [1,3]. CD133, also known as prominin-1, is a common CSC marker in patients with a poor prognosis and resistance to conventional therapies [3,10]. ALDH is a functional marker that is found at elevated levels in cells associated with the CSC niche. This enzyme is responsible for CSC chemoresistance and the detoxification of anti-cancer drugs by oxidizing aldehydes to carboxylic acids [1,10].

### 2.6. Signaling Pathways

CSCs use signaling pathways that are common with normal stem cells, namely, the Hedgehog, Notch, and TGF-β pathways. These pathways regulate stemness in many cancers [10]. Notch is an evolutionarily developmental pathway that plays an important role in cell-fate determination and tissue development. The Hedgehog pathway is involved in cell growth, migration, morphogenesis, and tissue maintenance and repair. TGF-β is an important prognostic marker for various types of cancer and plays a role in the initial phase of CSC development and self-renewal [1,3,10].

### 2.7. CYP Family of Enzymes

Cytochrome P450 (CYP) enzymes are involved in drug metabolism in the liver and small intestine. Their overexpression in the tumor tissue contributes to the degradation of anticancer drugs and multidrug resistance (MDR) [9]. The overexpression of CYP enzymes and specific biomarkers and the activation of stemness signaling pathways are used in targeting anticancer drugs to the CSC niche [7].

## 3. Polymer-Based NPs

Despite significant advances in targeted therapies, cancer patients suffer from relapse due to drug resistance and the persistence of CSCs in the tumor tissue. While conventional therapies are effective in eliminating bulk tumor cells, the small population of CSCs left behind undergo an asymmetric division to form new stem cells, as well as differentiated cells that repopulate the tumor tissue. Further, as most drugs are not specifically targeted to cancer cells or CSCs, they suffer from serious undesired side effects [29,30]. Consequently, there is a need to develop novel delivery systems to target cancer drugs to CSCs, the most resistant and invasive cells in the tumor tissue. Due to their size and ability to penetrate the dense tumor tissue, NPs serve as an attractive carrier for targeted drug delivery to tumors. Recently, many NP types, including self-assembled polymeric NPs, inorganic NPs, natural NPs based on proteins and exosomes, and antibody-drug conjugates, have been developed in an attempt to target chemotherapeutic agents to surface biomarkers, biomolecules in CSCs’ signaling pathways, or sites of overexpressed enzymes in the CSC niche. Drug-loaded NPs not only protect the cargo from enzymatic degradation and diffusion away from the target site, but they also improve the drug’s pharmacokinetics [2,10]. Aside from their high surface to volume ratio and unique optical properties, NPs improve the bioavailability of hydrophobic drugs in physiological media, increase drug stability, and allow for timed-release in the target tissue [7,10]. Moreover, NPs enable the concurrent targeted delivery of both hydrophilic and hydrophobic drugs to cells in bulk tumors, as well as to CSCs at a relatively high loading capacity [24,31]. The toxicity, bioavailability, and effectiveness of drugs loaded in NPs depend to a large extent on the physiochemical and biological properties of NPs, including the size and distribution, surface charge, hydrophilicity, drug release rate, pharmacokinetics, and other biochemical factors [10]. There are two main approaches to targeting chemotherapeutic agents to tumor cells using NPs:

1. Passive targeting: Pathophysiological conditions, specifically impaired angiogenesis and a high demand for nutrients and oxygen by proliferating tumor cells, result in an overexpression of vascular endothelial growth factor (VEGF) and the formation of abnormal tumor vessels, with relatively large gaps between the endothelial cells’ lining lumen of the vessels. The large intercellular clefts and poor lymphatic drainage leads to an accumulation and retention of NPs, with a size range of 100–200 nm in the tumor tissue. This enhanced permeability and retention (EPR) effect allows for the passive targeting of drug-loaded NPs to the tumor vasculature. However, NPs with a short circulation time are rapidly taken up by the mononuclear phagocyte system (MPS), prior to uptake by the tumor vasculature. Therefore, NPs should be surface modified to prolong their residence time in circulation [32,33]. The surface modification of NPs with non-adhesive, highly water soluble polymers, like polyethylene glycol (PEG), polyacrylic acid (PAA), and dextran, has been shown to reduce the undesired uptake of NPs by MPS [1].
2. Active targeting: Antibodies or ligands that interact specifically with one or multiple CSC surface biomarkers are used for targeting therapeutic agents to stem cells in the tumor tissue. This approach significantly reduces drug toxicity and undesirable uptake by normal cells [8].

Despite their many advantages as a carrier for drug targeting to CSCs, NPs are quickly cleared from the circulation, taken up passively by pinocytosis, cause pulmonary inflammation, translocate to other tissues, and tend to aggregate [11]. The use of drug-loaded NPs surface conjugated with multifunctional antibodies and ligands targeting concurrently to two or more biomarkers on CSCs can significantly reduce drug toxicity and side effects while improving effectiveness [8].

### 3.1. PLGA NPs

Poly(lactide-co-glycolide) (PLGA) is a biodegradable polymer used in many biomedical products and is the most frequently used carrier for preparing drug-loaded NPs [10]. In a study in nude mice with breast tumors, Yang et al. successfully used PLGA NPs, surface-modified with lipids, for the co-delivery of paclitaxel (PTX) and curcumin (CUR) [34]. CUR in PLGA NPs inhibited the growth of breast tumor cells by selectively targeting CSCs, while PTX eliminated bulk tumor cells. In a mouse breast tumor model, Li et al. conjugated Salinomycin (SLM)-loaded PLGA NPs with an antibody against erbB-2 tyrosine-protein kinase receptor (HER2) for targeting HER2-positive CSCs [35]. This approach inhibited tumor growth and reduced the CSC subpopulation of tumor cells in vitro and in vivo. In nude mice with ovarian tumors, PTX-loaded PLGA NPs conjugated with folic acid (FA) reduced the expression of chemo-resistant genes ABCG2 and MDR1 and increased the expression of apoptotic markers in tumor cells [36]. In a study with mouse CSCs with Kras mutation, the antimicrobial agent anthothecol encapsulated in PLGA NPs inhibited the migration and growth of pancreatic CSCs and induced apoptosis by modulating the sonic hedgehog pathway [37]. It also inhibited colony formation by human and mouse pancreatic CSCs in vitro. In another study in a Saos-2 osteosarcoma xenograft mouse model, SLM-loaded PEGylated PLGA NPs, surface modified with CD133 aptamer, eliminated CD133-positive osteosarcoma CSCs in vitro and in vivo [38]. In a study with MDA-MB-231 cells, PLGA NPs loaded with PTX and SLM and coated with hyaluronic acid (HLA) showed a high binding efficiency against CD44^+^ cells and cytotoxicity against both bulk tumor cells and CSCs [39].

### 3.2. PEG NPs

Polyethylene glycol (PEG) is widely used in medical applications to prevent protein adsorption on biomaterials and evade the immune system [10]. In one study, pH-sensitive PEG NPs were developed for the co-delivery of doxorubicin (DOX) and the antineoplastic drug, SN38, to tumor cells [40]. The systemic evaluation of metabolites showed an enhanced accumulation of the drugs in the tumor tissue through a passive EPR effect and the elimination of both bulk tumor cells and CSCs. In another study, SLM-loaded PEG-ceramide nano-micelles (SCM) showed toxicity toward both bulk liver tumor cells and CSCs [41]. In a study in a BP-474 human breast carcinoma xenograft mouse model, di-block self-assembled nano-micelles based on copolymers of PEG and acid-functionalized polycarbonate were used for the co-delivery of DOX and thioridazine (THZ) to eradicate bulk cancer cells and CSCs [42]. This combinational therapy is a promising approach for treating patients with metastatic breast cancer.

### 3.3. PLGA-PEG Copolymer NPs

Copolymers of PLGA and PEG that self-assemble into core-shell NPs have been used for drug delivery to CSCs. In one study, SLM-loaded PLGA-PEG NPs conjugated with antibody against the CD133 marker were used for targeting CD133-positive ovarian CSCs [43]. In a nude mouse model with ovarian tumor xenograft, the drug-loaded NPs showed an enhanced bioavailability of SLM and a reduction in the fraction of CD133-positive CSCs in the tumor tissue. In another study, a murine model of MDA-MB-231 orthotopic tumor was used to evaluate PLGA-block-PEG (PLGA-b-PEG) NPs loaded with docetaxel (DTXL) and small interfering RNA (miRNA) targeting BMI-1. BMI-1 is a member of the Polycomb repressor complex-1, which is implicated in CSC self-renewal by mediating gene silencing and regulating the chromatin structure [44]. The bulk tumor cells were eliminated by the release of DTXL, whereas the released miRNA downregulated the expression of the BMI-1 oncogene in the CSCs, which reduced the expression of the stemness markers and increased the sensitivity of CSCs to DTXL. Zhang et al. used a combination of SLM- and gefitinib-loaded NPs, synthesized separately by an emulsion-solvent evaporation approach, to selectively eliminate CD133^+^ CSCs in the spheroids of CD133^+^ lung cancer cells in vitro and in a xenograft mouse model inoculated with CD133^+^ lung tumor cells [45]. The combined delivery of SLM- and gefitinib-NPs was more effective in eliminating CD133^+^ CSCs and reducing tumor volume, compared to SLM/gefitinib-loaded NPs, or individually delivered SLM-NPs and gefitinib-NPs. It is possible that CSC targeting antibodies or ligands interact with normal stem cells (NSCs) to cause undesired cytotoxic effects, as CSCs share surface markers and signaling pathways with NSCs. In this regard, PLGA-PEG dual-targeted NPs surface conjugated with hyaluronic acid and doublecortin-like kinase-1 (DCLK1) monoclonal antibody against CD44 and DCLK1 cell surface receptors, respectively, for CSC targeting were evaluated for off-target toxicity [46]. The dual-targeted NPs discriminated between CSCs and NSCs in vitro, when tested with 4T1 CSCs in an alginate-based platform and in 4T1 inoculated nude mice in vivo. In another study, all-trans retinoic acid (ATRA)-loaded PLGA-lecithin-PEG NPs conjugated with CD44 and CD133 antibodies were more effective in inhibiting the growth of CSCs, compared to single-antibody-targeted NPs or non-targeted NPs [47].

### 3.4. Polylysine NPs

**Figure 2:**
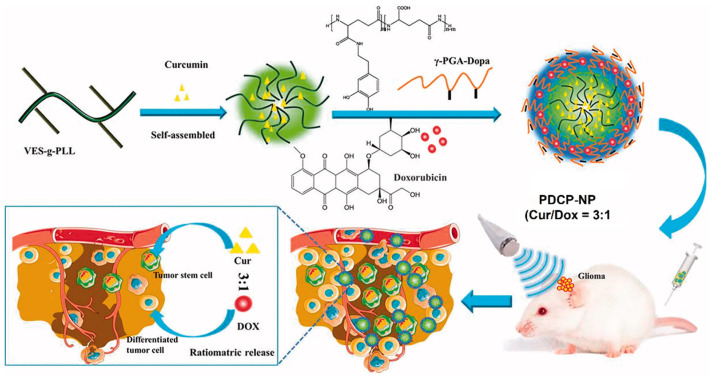
Schematic diagram showing the synthesis of pH-sensitive polylysine-polyglutamic acid core-shell NPs for the co-delivery of DOX and CUR to the bulk of tumor cells and CSCs in glioma. Adapted from [48] under the terms of the Creative Commons CC BY license.

The co-delivery of hydrophilic DOX and hydrophobic CUR to brain tumors with an optimal dose ratio is limited by differences in the pharmacokinetics (PK) and blood brain barrier (BBB) properties of the two drugs. Xu et al. synthesized pH-sensitive core-shell polylysine-polyglutamic acid NPs for the co-delivery of DOX and CUR [48]. The hydrophobic CUR was encapsulated in the tocopheral-grafted polylysine core, whereas the hydrophilic DOX was encapsulated in the anionic dopamine-modified polyglutamic acid shell deposited on the core via a pH-sensitive linkage (Figure 2).

DOX and CUR encapsulated in the core-shell NPs were effectively delivered in a predefined ratio to the bulk of tumor cells and CSCs in the glioma, respectively, in vivo in C6-inoculated rat glioma, which decreased the fraction of CSCs from 4% to <1%. These core-shell NPs are promising as a carrier in combination therapies for the delivery of cancer drugs with dissimilar physiochemical properties [48]. In another study, the tumor suppressor microRNA-34a implicated in epithelial to mesenchymal transition (EMT) was loaded in imidazole-grafted poly-L-lysine NPs coated with PEGylated lipids to reduce toxicity and improve stability in blood circulation by neutralizing the surface charge [49]. The microRNA-34a-loaded NPs evaluated in a mouse model of gastric tumor showed the inhibition of CSC migration and tumor formation, induced apoptosis, and eliminated the CSC subpopulation of tumor cells by suppressing the CD44 expression.

### 3.5. PLA-PEG NPs

PEG is copolymerized with biocompatible and biodegradable polylactide (PLA) to produce self-assembled NPs [10]. In one study, PLA-PEG NPs were used for the co-delivery of DOX and chloroquine (CQ) to eliminate bulk tumor cells and CSCs in ALDH^+^ MDA-MB-231 breast cancer cells [50]. In another study, PLA-PEG NPs, produced by a single emulsion method, were loaded with DOX and ATRA, a differentiation agent for CSCs. This combinational drug delivery system markedly increased the concentrations of DOX and ATRA in the tumor tissue and synergistically suppressed tumor growth [51]. DNA repair plays an important role in the self-renewal and maintenance of CSCs. The DNA hypermethylation inhibitor decitabine (DAC) encapsulated in PLA-PEG NPs and combined with DOX-loaded PLA-PEG NPs significantly downregulated the expression of enzymes that catalyze DNA methylation (DNMT1 and DNMT3) in an MDA-MB-231 xenograft murine tumor model [52]. Further, this dual-delivery system increased the sensitivity of bulk tumor cells and CSCs to DOX, which led to a reduction in tumor burden in breast cancer.

### 3.6. Lipid-Polymer NPs

**Figure 3:**
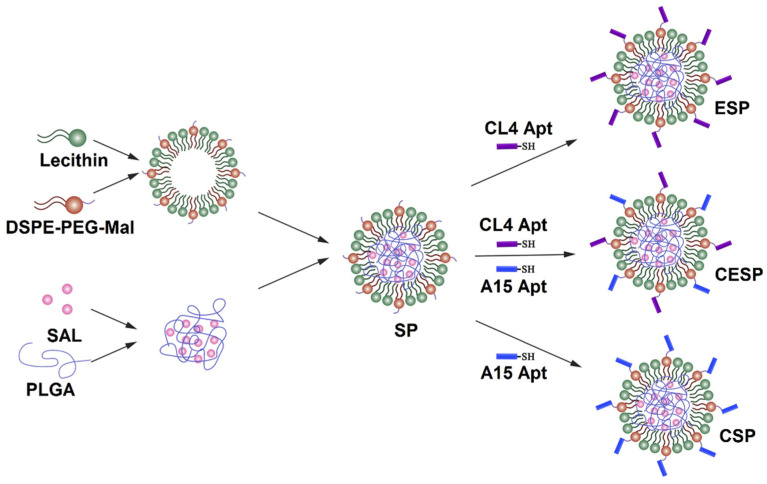
Schematic diagram of the preparation of SLM-loaded LP NPs conjugated with anti-EGFR (ESP), anti-CD133 (CSP), and both anti-EGFR and anti-CD133 (CESP) aptamers. Reprinted with permission from ref. [55], Copyright 2018 Elsevier.

Lipid-polymer (LP) NPs consist of a polymeric core enveloped by a lipid shell. The lipid shell is used for the conjugation of antibodies against cell surface receptors on tumor-associated cells for the targeted delivery of cancer drugs [53]. In one study, SLM-loaded NPs, prepared by a single-step nanoprecipitation method, were surface conjugated with anti-CD20 aptamers via a maleimide-thiol reaction for targeting melanoma CSCs [54]. CD20-positive melanoma cells showed a higher uptake of SLM-loaded NPs in vitro, compared to the NPs without the aptamer conjugation or free SLM. In another study, SLM was targeted to bulk tumor cells and CSC in osteosarcoma tumor model by encapsulation in LP NPs conjugated with anti-CD133 and anti-EGFR aptamers [55]. Three types of LP NPs were used in this study, as shown in Figure 3, which included SLM LP NPs conjugated with anti-EGFR aptamer (ESP); SLM LP NPs with anti-CD133 aptamer (CSP); and SLP LP NPs with both anti-EGFR and anti-CD133 aptamers (CESP). The results in an osteosarcoma mouse model showed a higher effectiveness of CESP NPs in targeting both bulk tumor cells and CSCs and inhibiting tumor growth.

### 3.7. mPEG NPs

**Figure 4:**
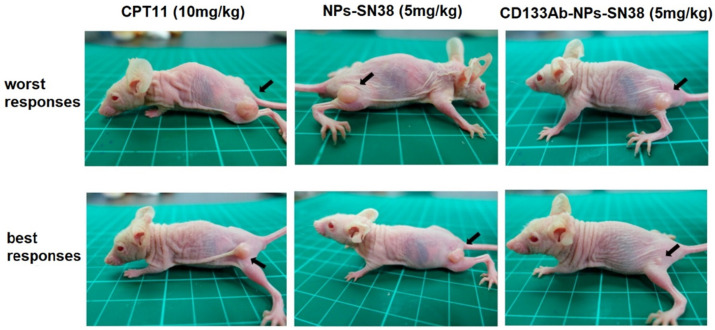
Comparative illustration of the tumor size in a HCT116 xenograft mouse model treated with CPT11 control group, SN-38 NPs, and CD133Ab-SN38 NPs, adapted from. Reprinted with permission from ref. [58], Copyright 2016, American Chemical Society.

Block copolymers of polyethylene glycol methyl ether (mPEG) and poly(diethyl disulfide) have been used to produce glutathione (GSH)-responsive micelles, reactive oxygen species (ROS)-responsive micelles, and dual GSH-/ROS-responsive micelles for tumor-specific drug delivery [56]. DOX and STAT3, a signal transducer inhibitor and activator of transcription-3, were loaded in mPEG-based GSH-/ROS-responsive micelles and evaluated for toxicity toward initiating CSCs (ICSC) and metastatic CSCs (MCSC) with colon cancer cells. The dual GSH-/ROS-responsive micelles eliminated both ICSCs and MCSCs, whereas GSH-responsive micelles had no effect on MCSCs. This was attributed to the low GSH expression of MCSCs. In another work, PTX and hedgehog-inhibitor cyclopamine (CPY) were conjugated to copolymers of mPEG and poly(2-methyl-2-carboxyl-propylene carbonate) (PCC) as a combinational therapy for prostate cancer [57]. The results from in vivo experiments showed an increased concentration of PTX/CPY-conjugated NPs in the tumor tissue from a passive EPR effect. Further, dual PTX/CPY-conjugated NPs showed a higher inhibition of tumor growth, compared to PTX- or CPY-conjugated NPs. In another work, mPEG-polycaprolactone (mPEG-PCL) NPs conjugated with an antibody against CD133 receptors were used for the delivery of a topoisomerase inhibitor (SN-38) to CD133-positive cells in an HCT116 xenograft mouse model [58]. The SN-38 loaded, anti-CD133 conjugated NPs showed a higher suppression of tumor growth and reduction of tumor size, compared to SN-38-loaded NPs without anti-CD133 or direct treatment with irinotecan (CPT11) (Figure 4).

### 3.8. Hyaluronic Acid NPs

Hyaluronic acid (HA) is an anionic, non-sulfated glycosaminoglycan found in epithelial, connective, and neural tissues. The thiolated HA (HA-SS) is sensitive to glutathione. Dual sensitive HA-SS conjugated with 6-mercaptopurine (MP) for targeting CD44 receptors on tumor cells were used for targeting DOX to CSCs in colon cancer therapy [59]. The DOX-loaded HA-SS-MP NPs showed a higher drug release at pH 5 in the presence of GSH than the physiological pH 7 and a higher uptake by cancer cells with an upregulated CD44 expression. The studies in an HCT116 xenograft mouse model showed a higher inhibition of tumor growth by DOX-loaded HA-SS-MP NPs, compared to the free drug. The reduction in tumor size in the HA-SS-MP NP group was attributed to the higher DOX concentration in the tumor tissue. In a recent study, HA-coated NPs loaded with docetaxel (DTX) and photosensitizer meso-tetraphenyl chlorine disulfonate (TPCS) showed a growth inhibition of breast cancer CSCs, compared to HA-mediated monotherapy [60].

### 3.9. PLGA/TPGS NPs

NPs based on a mixture of PLGA and d-α-Tocopheral Polyethylene Glycol 1000 Succinate (TPGS) were used by Chen et al. for the co-delivery of DOX and elacridar (ELC) to liver cancer cells [44]. Based on in vitro studies and in vivo results from an HepG2 xenograft mouse model, the optimum DOX/ELC ratio was 1:1 for optimum tumor targeting and the inhibition of tumor growth. Chen et al. also showed that the optimum DTX to SLM ratio was 1:1 for the suppression of tumor growth in a breast tumor model [61].

### 3.10. Liposomes

Liposomes are amphiphilic phospholipid vesicles used for the delivery of both hydrophilic and hydrophobic drugs [4]. A novel redox-responsive liposome was developed for the co-delivery of SLM and DOX to liver CSCs by targeting CD133 and epithelial cell adhesion molecule (EpCAM) receptors on the surface of CSCs [62]. According to the experimental results, these liposomes were endocytosed by CSCs and degraded in the cytoplasm for the rapid intercellular release of SLM and DOX to synergistically inhibit CSC growth.

In another work, dual-targeting cationic liposomes with specificity to CD133^+^ glioma stem cells were synthesized by conjugation with a low-density lipoprotein receptor-related protein and an RNA aptamer targeting CD133 receptor on CSCs [63]. PTX and survivin siRNA loaded in the dual-targeting liposomes induced the differentiation of glioma CSCs and tumor cell death in a U251-CD133^+^ glioma xenograft mouse model. There is a need to develop targeted therapies for patients with triple-negative breast cancer (TNBC), as the expressions of estrogen receptor (ER), progesterone receptor (PR), and human epidermal growth factor receptor-2 (HER2) in TNBC are downregulated. Multifunctional liposomes (MLPs) surface-modified with chitosan, loaded with gambogic acid (GA), and labeled with Zirconium-89 (^89^Zr) have been developed for targeting CD44^+^ CSCs in TNBC [64]. Micro positron emission tomography (micro-PET) and Fluorescence imaging showed an accumulation of the MLPs in the tumor tissue and uptake by cells overexpressing CD44^+^ marker in an MDA-MB-231 xenograft mouse tumor model. A comprehensive review of the use of liposomes in targeting tumor cells can be found elsewhere [4].

### 3.11. Multi-Polymeric NPs

Polymeric biomaterials, with their wide range of properties, are extensively used in targeted cancer therapies. A pH-sensitive PEG-benzoicimine-poly(γ-benzyl-L-aspartate)-b-poly(1-vinylimiazole) block copolymer (PPBV) was used to deliver CUR and PTX drugs to CSCs and bulk tumor cells [65]. A unique feature of PPBV is its ability to switch its surface charge from neutral to positive, de-shield its PEG layer, and reduce its size to penetrate deep into the tumor tissue. In another study, thermally-responsive and pH-sensitive NPs based on Pluronic F127, PLGA, and chitosan coated with hyaluronic acid (HA) were used to target DOX and irinotecan (Camptosar or CPT) drugs to bulk tumor cells and CSCs in a prostate xenograft mouse model [66]. The HA coating of the NPs was used to target the drugs to CSCs in the prostate tumor tissue. The HA coating replaced polyvinyl alcohol (PVA) as a stabilizer in the double emulsion method used to form the NPs. Based on the in vitro and in vivo results, the DOX and CPT loaded NPs synergistically reduced the drug resistance of CSCs in the prostate tumor tissue.

### 3.12. Other Polymeric NPs

Cationic albumin NPs loaded with ATRA and functionalized with HA have been developed for targeting the CD44 receptor in CSCs [67]. In vivo imaging in a mouse model showed an accumulation of the NPs in a tumor-bearing lung and the suppression of tumor growth. Photodynamic therapy (PDT) generates cytotoxic singlet reactive oxygen species (ROS) in tumor tissue by transferring energy from a photosensitizer to the surrounding oxygen molecules. However, this approach is limited by the low levels of oxygen in the hypoxic environment of tumors. NPs based on sodium alginate and docusate loaded with a photosensitizer-like methylene blue have been used to overcome this limitation of PDT [68]. The experimental results with the methylene blue-loaded NPs demonstrated that the extent of ROS generation depended on the interaction of the cationic photosensitizer with the anionic alginate. The methylene blue-loaded NPs eliminated the CSCs in MCF7 tumor cells treated with PDT. In another work, Dox-loaded Pluronic F127 NPs surface-modified with chitosan were used as a pH-sensitive carrier for targeting CSCs [69]. The role of chitosan was to target the NPs to CD44^+^ cells and release the drug in the slightly acidic environment of the tumor. The results showed a 6-fold increase in the tumor toxicity of DOX-loaded, pH-sensitive, and CD44^+^-targeting NPs, as compared to free DOX. In another study, silk fibroin nanogels, formed by an aqueous process, were used as a carrier for targeting SLM and PTX to CSCs and bulk tumor cells in a hepatic tumor mouse model [70]. The nanogel carrier showed effectiveness against bulk tumor cells and CSCs in vivo. In another study, the addition of *N*,*N*-Dimethylhexylamine (DMHA) and α-tocopheral additives to immune-tolerant, elastic-like polypeptide (iTEP) NPs loaded with SLM improved the loading efficiency and half-life of the NPs in circulation by 4-fold [71]. Further, the additives increased the area-under-curve (AUC) for SLM in the plasma by ten times, which increased the accumulation of SLM in the tumor tissue and enhanced the elimination of CSCs in a 4T1 orthotopic tumor model.

Polymeric NPs represent an ideal platform for targeted drug delivery to CSCs because of their biodegradability, biocompatibility, and storage stability. However, the particle aggregation and toxicity can be limiting factors in the long term. As a result, only a few polymeric NPs have been approved by the FDA for clinical use [72].

## 4. Inorganic NPs

Inorganic NPs, with sizes as low as a few nanometers and a uniform distribution, are very attractive as passive or active carriers in tumor targeting [73]. However, their use is limited by their rapid clearance from circulation through the reticuloendothelial system (RES), as well as their rapid recognition and elimination by scavenger receptors on Kupffer cells in the liver [74,75]. These limitations can be overcome by tailoring their physicochemical properties, such as particle shape, size, and surface chemistry, to a specific cancer therapy application. It should be noted that each type of inorganic nanomaterial possesses its own unique properties, which determine its interaction with cells, particularly with respect to cell uptake.

### 4.1. Gold NPs

**Figure 5:**
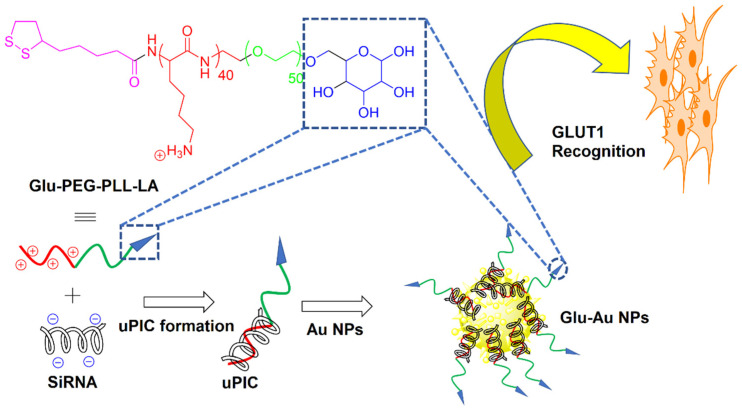
Schematic diagram of the two-step bottom-up approach for the synthesis of self-assembled Glu AU NPs. Reprinted with permission from ref. [81], Copyright 2014 American Chemical Society.

Due to their biocompatibility, nontoxicity, and narrow size distribution, bare and surface-modified gold (Au) NPs have received considerable attention as passive or active drug carriers in cancer therapy [26,76]. The surface modification of Au NPs via ligand immobilization approaches improved their in vivo performance as a carrier for targeted tumor drug delivery [77]. Further, the surface functionalization of Au NPs with biocompatible coatings has improved their biocompatibility in physiological media. In one study, bare Au NPs were synthesized by sodium citrate reduction, followed by coating with thiol-terminated PEG, to form NPs with an average size of 20 nm [78]. PEGylation not only reduced the aggregation of Au NPs, but it also increased their stability and biocompatibility. The conjugation of SLM to PEGylated Au NPs improved the drug uptake by breast CSCs expressing CD24^−^/CD44^+^ markers and enhanced drug-induced tumor cell death. As cancer cells have a higher consumption of glucose (Glu) than healthy cells, Glu could potentially be used as a reagent in tumor targeting [79]. Recently, a two-step bottom-up approach was used to synthesize Glu Au NPs with an average size of <50 nm, as shown in Figure 5 [80]. In the first step, a Glu-functionalized PEG-block-cationomer was neutralized with a single pair of siRNAs by charge-matching to form unimer polyion complexes (uPICs). Next, the synthesized uPICs were immobilized on the surface of Au NPs by Au-S coordination between the AU surface and thiol groups of Glu to form monodisperse Glu-Au NPs. The Glu moieties on the surface of Au NPs led to their recognition by glucose transporter-1 (GLUT1) overexpressed on the surface of CSCs. The Glu functionalized Au NPs improved the antitumor activity toward GLUT1-overexpressing MDA-MB-231 spheroids and MDA-MB-231 orthotropic tumors. The same group previously reported the synthesis of two other uPIC-Au NPs as a carrier for the systemic delivery of siRNA to solid tumors [81,82].

Au NPs have also been functionalized with folic acid, transferrin, and bombesin peptides for targeted drug delivery to ovarian, prostate, and breast cancers, respectively [83,84,85]. Latorre et al. synthesized albumin-stabilized Au NPs by incubating gold salt with bovine serum albumin (BSA) in a basic medium, followed by the conjugation of DOX and SN38 topoisomerase inhibitor to the surface of NPs through disulfide and maleimide linkers, respectively [86]. The results showed a reduction in the number and size of MCF7 tumor spheroids after treatment with DOX/SN38-conjugated BSA-Au NPs.

### 4.2. Iron Oxide NPs

**Figure 6:**
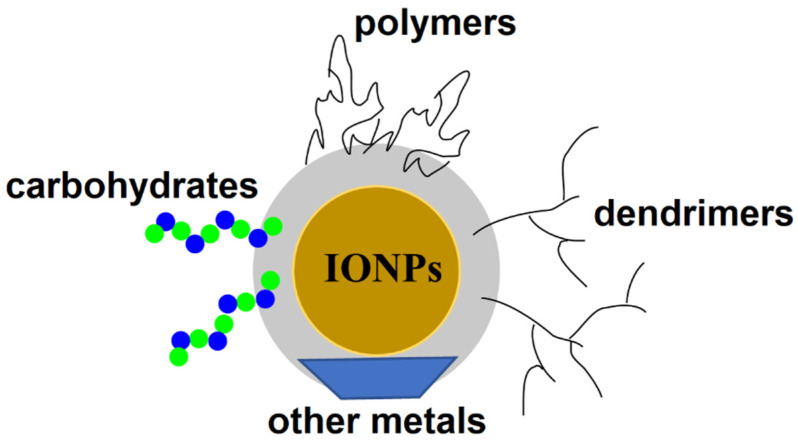
Schematic illustration of the surface functionalization of IONPs with various biomaterials, including polymers, dendrimers, carbohydrates, and other metals.

Magnetic NPs have been widely used in the treatment and diagnosis of various cancers. Among them, magnetic iron oxide nanoparticles (IONPs) possess unique properties, including non-toxicity, biocompatibility, and a high efficiency of drug and gene delivery to the target site [87]. These and other properties, such as an ease of surface functionalization, high colloidal stability in physiological media, excellent drug binding, and feasible large-scale production, make IONPs powerful nanocarriers for drug delivery to CSC subpopulations of cancer cells. IONPs have been coated with various biomaterials, including oligomers, dendrimer, carbohydrates, and polymers, as well as inorganic materials, to improve the efficiency of drug delivery to cancer cells (Figure 6).

Su et al. synthesized super-magnetic Fe_3_O_4_ NPs (SPIONPs) using a coprecipitation method in the presence of Fe^2+^ and Fe^3+^ in a basic medium [88]. Following the synthesis, the surface of SPIONPs were functionalized with carboxyl groups by a reaction with carboxymethyl dextran. The surface carboxyl groups were then used to covalently link a the CD44 antibody to SPIONPs via 1-ethyl-3-(3-dimethylaminopropyl)carbodiimide (EDC) chemistry by a reaction with the amine groups of the antibody. The magnetic fluid hyperthermia generated by the anti-CD44-functionalized SPIONPs in an alternating magnetic fluid (AMF) appreciably reduced the CSC subpopulation of tumor cells in a head and neck squamous cell carcinoma model. IONPs with multiple targeting modalities enable the binding of the NPs to multiple receptors on the surface of the CSCs in tumor cells. In this regard, ultra-small IONPs with an average size of 5 nm, surface-modified with two peptides targeting Wnt/LRP5-6 and the urokinase plasminogen activator receptor (uPAR), downregulated Wnt/β-catenin signaling and marker expression of CSCs, resulting in a greater inhibition of tumor growth in a patient-derived xenograft (PDX) breast tumor model, as compared to single-targeting IONPs [89].

### 4.3. Silica NPs

The use of silica NPs in drug and gene delivery has been growing in the last decade [90]. Mesoporous silica (MS) NPs as a drug carrier possess unique properties, including a tunable size, large surface area and porosity, ease of functionalization, and an ordered porous structure for drug delivery to CSCs [91]. Surface-functionalized MS NPs with defined shape and controlled pore size have been synthesized to efficiently deliver hydrophobic anticancer drugs and nucleic acids to tumors. Recently, a pH-responsive carrier was developed based on MS NPs conjugated with dendritic polyglycerol (dPG) for the co-delivery of DOX and tariquidar (TAR) to the CSC subpopulation of breast cancer cells [92]. The DOX- and TAR-loaded MS NPs suppressed the expression of CSC-associated markers and blocked spheroid formation in a breast MDA-MB-231 spheroid model. In another work, MS NPs modified with cationic polyethyleneimine (PEI) were used as a dual-targeted carrier for the delivery of HNF4α-encoding plasmid and cisplatin to CSCs in hepatocyte-derived Huh7 carcinoma cells [93]. Based on the experimental results, the load MS NPs blocked the division of Huh7 cells in the S-phase of the cell cycle, leading to apoptosis.

Due to their magnetic, radioactive, and plasmonic properties, inorganic NPs are used clinically in diagnostic and imaging applications, as well as photothermal therapies. While most inorganic NPs possess a good biocompatibility and stability, their clinical applications are somewhat limited by their toxicity and low degradability in physiological media [72].

## 5. Self-Assembling Protein NPs

Conventional approaches to cancer treatment are limited by undesired toxic side effects and a lack of control over the local drug concentration in the tumor tissue, which has led researchers to explore alternative solutions. While nanocarriers improve the drug biodistribution and passive and active targeting, reduce renal clearance, protect the drug from degradation, and enhance cell uptake, only a fraction of the administered drug reaches the tumor tissue. Further, the persistence of the carrier in the tumor and healthy tissues leads to undesired toxic effects [94,95]. NPs based on multifunctional proteins that are degraded by natural enzymatic pathways are attractive as a carrier for passive or active drug targeting to tumors [96]. CXCR4 is a viable target in cancer therapy, because it mediates cancer metastasis by inducing the migration of tumor-associated cells. A single-chain variable fragment (scFv) antibody targeting CXCR4 was fused with an RNA-binding protein peptide (RBM) and mixed with miR-127-5p, a mediator of M1 macrophage polarization, to form self-assembling RNA-protein nanoplexes [97]. These nanoplexes served as a carrier for targeting miRNA to tumor-associated cells that express CXCR4. In a 4T1 TNBC mouse model, these nanoplexes inhibited the migration of tumor-associated cells, polarized the macrophages to the M1 phenotype, and suppressed tumor growth [97]. In another study, a modular fusion protein composed of an N-terminal cationic peptide T22-targeting CXCR4 receptor on tumor cells and a C-terminal polyhistidine tag (H6) on a fluorescent GFP protein scaffold for imaging was used to form self-assembled NPs for tumor targeting [98]. The peptide T22 and polyhistidine tag H6 induced the self-assembly of the modular protein into fluorescent NPs with an average size of 12 nm. The T22 peptide facilitated the binding and internalization of the NPs in CXCR4^+^ tumor cells for targeted intracellular drug delivery. In a recent study, the drugs, oligo-floxuridine (FdU) and monomethyl auristatine E (MMAE), were chemically coupled to exotoxin A from Pseudomonase aeruginosa and diphtheria toxin from Corynebacterium diphtheria, respectively, to form self-assembled protein NPs with an average size of 50 nm targeting CXCR4^+^ tumor cells [95]. Based on in vitro studies, the resulting protein NPs were internalized by CXCR4^+^ cells and inhibited the growth of tumor cells. Ribosome-inactivating proteins (RIPs) are considered potent therapeutic agents for cancer therapy, as they inactivate ribosomes in cancer cells and inhibit protein synthesis, leading to cell death. In this regard, magnetic NPs were surface modified with a fusion protein composed of the small protein, Barstar (Bs), synthesized by Bacillus amyloliquefaciens, which inhibits bacterial ribonuclease and the C-terminal part of the magnetite binding protein of magnetotactic bacteria (Mms6) [99]. These Bs-C-Mms6 magnetic NPs undergo a spontaneous self-assembly with a Barnase-containing biomolecule by a specific Barstar-Barnase interaction for targeted drug delivery. As a proof of concept, a fusion protein of Barnase and the peptide DARPin9.29 that binds to the HER2/neu receptor underwent a self-assembly with Bs-C-Mms6 NPs to target magnetic particles to HER2/neu overexpressed cells in breast cancer tissue [99]. Gelonin is a ribosome-inactivating protein (RIP) used in cancer therapy to block the growth of cancer cells. In one study, gelonin was conjugated to monocrystalline nickel-iron oxide (NiFe_2_O_4_) NPs (MIONs) using a multifunctional peptide linker for targeted delivery to tumor cells in a fibrosarcoma xenograft mouse model [100]. The multifunctional peptide consisted of a 6-mer histidine tag (6His-Tag) for attachment to the MION followed by a matrix metalloproteinase-2 (MMP-2) degradable sequence and a low-molecular-weight peptide (LMWP) for cell penetration. Following uptake, the MMP-2 degradable peptide is degraded by overexpressed MMP-2 in the tumor tissue, resulting in the release of gelonin-LMWP and endocytosis by the tumor cells, facilitated by the cell penetrating peptide. The in vivo results showed an enhanced cytotoxicity of the MIONs against the tumor cells in a fibrosarcoma xenograft mouse model [100]. These studies indicate that protein NPs, due to their biodegradability and tunable self-assembly, are especially useful for the delivery of amino acid-based bioactive agents, such as RIPs and antibodies.

Naturally occurring or synthetic amino acid sequences used in assembling protein NPs can be immunogenic. The immune response can neutralize the drug’s effectiveness or cause serious side effects in therapeutic applications. In some cases, these peptides can be immunosuppressive, and their long-term administration can cause severe side effects, such as relapsed bacterial, viral, or fungal infections [101]. The targeting agent in the delivery of cytotoxic proteins should have a high selectivity for receptors on tumor-associated cells to reduce the risk of serious side effects in healthy tissues [102].

## 6. Antibody Drug Conjugates

**Figure 7:**
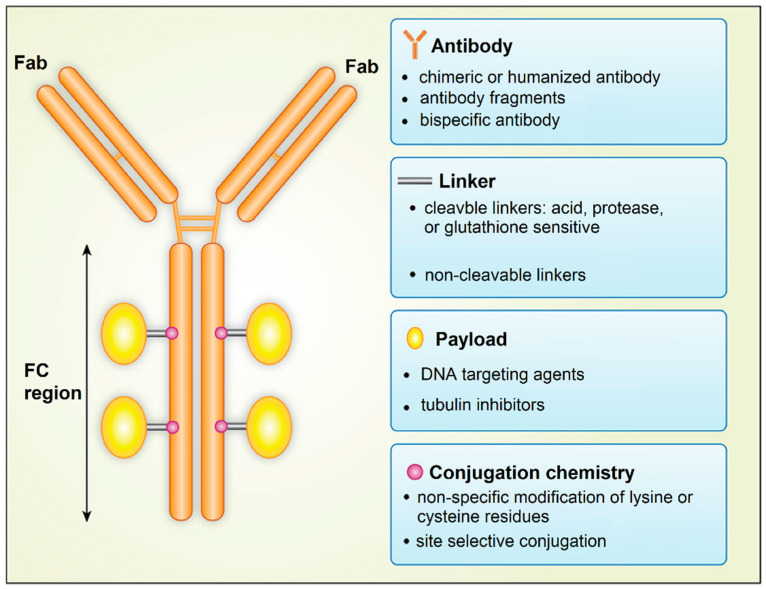
Schematic diagram showing the structure of the antibody drug conjugate. Adapted from [106] under the terms of the Creative Commons CC BY license.

An exciting approach to the targeted delivery of drugs in cancer therapy is the use of antibody-drug conjugates (ADC), which was named as the “magic bullet” by Paul Ehrlich [103]. He proposed the use of an antibody against tumor cells conjugated to the diphtheria toxin for cancer therapy. ADCs are drugs designed to target specific receptors on tumor cells, CSCs, or tumor-associated cells for localized intracellular delivery [104,105]. An ADC consists of a drug bound to an antibody by conjugation via a special protein, called the linker protein (Figure 7). After binding to a surface receptor on CSCs, ADC is engulfed by the CSC, and the drug is released from the conjugate to eliminate the cell [104]. The most important factor in the design of an ADC is the selection of a target antigen that binds with a high specificity to the antibody to minimize off-target toxic side effects to healthy cells. The number and density of target antigen molecules on the surface of CSCs affect the extent of the ADC engulfment by tumor cells. Further, antigen secretion by the CSCs to the circulation should be minimal to limit the circulatory detection of the ADC-antigen and activation of the immune system. Table 1 summarizes the list of surface antigens on tumor cells used in designing antibody-drug conjugates for different types of cancers [104,106].

Another factor is the selection of an antibody to target the antigens on the surface of CSCs with a high specificity, strong binding affinity, and long circulation half-life for prolonged uptake and retention by the tumor tissue. The efficient internalization by CSCs and low immunogenicity of the ADC are important factors in the selection process [106,107]. The pharmacokinetics and pharmacodynamics of ADC is affected by the choice of linker that connects the antibody to the drug molecule. The linker should stabilize the ADC in circulation but provide a mechanism for the release of the payload when the ADC reaches the tumor site. Linkers are divided into non-cleavable and cleavable linkers [108]. Non-cleavable linkers form stable bonds with antibodies and have a longer half-life in circulation. Further, after internalization, non-cleavable linkers should degrade in the lysozyme to release the drug molecule inside the targeted tumor cell. The stability of cleavable linkers depends on the physiological conditions in the tumor tissue and the expression of enzymes for cleavage of the linker to release the drug in the tumor site. The tumor toxicity of the ADC depends on the choice of payload (drug molecule) attached to the antibody. Conventional cancer drugs, such as DOX or mitomycin, have been used with ADCs. In general, the payload in ADCs is engineered to target either DNA or tubulin to interfere with cell division and proliferation. The conjugation chemistry affects the drug pharmacokinetics and therapeutic index of ADC. The conjugation of the payload largely occurs through the linker via the alkylation or acetylation of lysine side chains in the backbone of the antibody [106].

**Figure 8:**
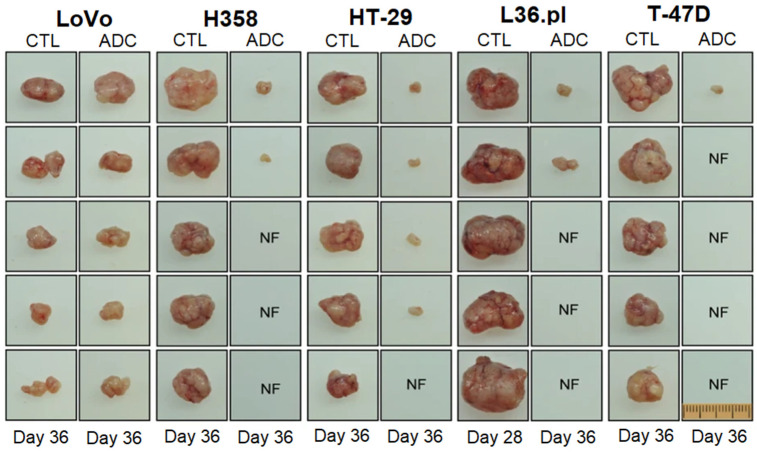
The effect of anti-RON ADC in the inhibition of tumor growth in LoVo colorectal, H358 non-small cell lung, HT-29 colon, L36.pl pancreatic, and T-47D breast xenograft tumor models. The control (CTL) was xenograft tumors mediated by LoVo cells. NF refers to no observed tumor. Adapted from [114] under the terms of the Creative Commons CC BY license.

ADCs possess a higher tumor selectivity and cytotoxicity, compared to other targeted delivery approaches [103]. In a recent study, natural phospholipids were mixed with PTX and SLM conjugated to monoclonal antibody 2C5 to form PTX/SLM-ADC immunoliposomes for targeted delivery to bulk tumor cells and CSCs in TNBC. The in vitro studies with MDA-MB-231 TNBCs and HER2-positive SK-BR-3 breast cancer cells showed a specific uptake of PTX-ADC and SLM-ADC by bulk tumor cells and CSCs, respectively [109]. Tumor-differentiation antigen or mesothelin, derived from tumor proteins, are overexpressed in ovarian, pancreatic, and lung cancers, as well as mesothelioma [110]. In one study, the maytansinoid tubulin inhibitor, DM4, was targeted to mesothelioma, pancreatic, and ovarian tumors overexpressing mesothelin by conjugation to an anti-mesothelin antibody via a disulfide-containing linker [111]. The in vitro studies showed a selective uptake of the ADC by mesothelin-expressing cells, depending on the expression level of mesothelin, without affecting mesothelin-negative cells, whereas the in vivo studies in a xenograft model showed the localized delivery of the ADC to mesothelin-positive tumors and inhibition of tumor growth [111]. In another work, an ADC based on a novel topoisomerase I inhibitor conjugated with an anti-HER2 antibody using a peptide linker was developed to treat trastuzumab-emtansine (T-DM1)-insensitive, high HER2-positive breast cancers [112]. The in vivo results revealed that the ADC was well tolerated at high doses in cynomolgus monkeys, and it was effective in a T-DM1-insensitive, high HER2 expressing PDX model. Further, the ADC also showed an antitumor efficacy in a breast PDX tumor model with a low HER2 expression, in which T-DM1 was not effective [112]. The RON tyrosine kinase receptor on macrophage-stimulating proteins (MSP) and its PSI domain, which facilitates the proper positioning of RON for ligand-receptor binding, are implicated in tumor progression in TNBC [113]. In one study, antineoplastic monomethyl auristatin E and duocarmycin DNA-alkylating agents were conjugated to the humanized antibody against the RON PSI domain as an anti-RON ADC [114]. The ADC inhibited the spheroid formation and eliminated the CSCs with the RON^+^/CD44^+^/ESA^+^ phenotype. A single injection of the ADC inhibited the tumor growth in multiple xenograft tumor models, including LoVo colorectal, H358 non-small cell lung, HT-29 colon, L36.pl pancreatic, and T-47D breast cancers, as shown in Figure 8 [114].

A target antigen in acute myeloid leukemia (AML) is the myeloid differentiation antigen, CD33 [115]. A DNA-alkylating antitumor agent was conjugated to a humanized anti-CD33 antibody using sulfo-N-Succinimidyl 4-(2-pyridyldithio)-butanoate to form a CD33-targeting ADC for the treatment of acute myeloid leukemia (AML) [116]. The in vitro cytotoxicity studies of the anti-CD33 ADC showed DNA damage, cell-cycle arrest, and apoptosis against patient-derived AML cells, whereas the in vivo studies in an AML xenograft model showed tumor regression and a prolonged survival [116]. The sialyl-thomsen-nouveau (STn) carbohydrate is attached to protein surface markers, such as the CD133 of CSCs in pancreatic, colon, gastric, and ovarian cancers [117]. An ADC based on an anti-STn antibody conjugated to antineoplastic drug monomethyl auristatin E (MMAE) eliminated STn^+^ ovarian cancer cells in vitro and reduced the tumor volume by the depletion of STn^+^ CSCs in an ovarian tumor xenograft model [118].

These studies clearly demonstrate that with the proper selection of an antibody specifically targeting surface receptors on CSCs, the appropriate choice of a therapeutic agent and suitable selection of a linker that degrades enzymatically to release the drug intracellularly in CSCs, the ADC approach is highly effective in eradicating highly malignant, metastatic, and recurrent cancers. Future studies should focus on the limited expression of antigens expressed exclusively on CSCs, linker stability, incomplete ADC internalization, and insufficient effectiveness of cancer therapeutic agents.

One of the challenges in designing ADCs for clinical applications is the stability of the linker. ADCs can circulate in the bloodstream for a significant amount of time, before reaching their target tissue. Therefore, the linker should have a sufficient stability to prevent the premature release of the cytotoxic agent in circulation [103]. This is a serious concern, because most cancer drugs used with ADCs are highly toxic in their free form. As a result, the premature release of the drug in circulation can cause serious side effects, such as low red and white cell counts, low platelet count, damage to the liver, peripheral neuropathy, and vision problems [104]. Conversely, a proper linker selection in ADCs ensures the targeted delivery of cytotoxic drugs to CSCs in the tumor tissue with a high selectivity [107].

## 7. Extracellular Vesicles

Extracellular vesicles (EVs) are lipid bilayer NPs exocytosed by cells for the intercellular transport of biomolecules and cell signaling. The diameter of EVs is in the range of 30–2000 nm, and these vesicles contain proteins, nucleic acids, lipids, and sugars. In cancer, EVs contribute to tumor progression by transporting and sharing biomolecules that enhance tumor growth or resist therapy between cancer cells [119]. Exosomes are formed in the cytoplasm and have a diameter of 30–150 nm, whereas microvesicles are formed by the outward expansion and fission of the cell membrane and have a diameter of 200–2000 nm. Apoptotic bodies, which are the largest EVs with a diameter of 500–2000 nm, are released during apoptosis and contain a cell nucleus, organelles, and proteins from the apoptotic cell [120]. In cancer, EVs have attracted interest for reversing tumor progression, because they facilitate cell–cell communication and maintenance and elicit a constructive, as opposed to inflammatory, immune response by acting as antigen-presenting vesicles [121]. EVs exchange information between CSCs and other tumor cells in the form of functional proteins, mRNAs, miRNAs, and small DNA fragments that contribute to tumor growth and progression. Cancer cells release more exosomes than other cell types to regulate the metabolism of the recipient cells, reprogram the cells to undergo apoptosis, mitosis, or angiogenesis, suppress the response of immune cells, or transfer oncogenic factors. Exosomes released by CSCs could transport stemness-associated factors or drug-resistant factors to the recipient cells [119]. Exosomes, due to their biocompatibility, low immunogenicity, long circulation time, and high loading capacity have been used as a nano-carrier in drug and gene delivery [122]. Further, the uptake of exosomes by tumor cells is >10-fold higher than that of liposomes, with a higher specificity of exosomes to tumor-associated cells [123]. Other EV types, such as microvesicles and apoptotic bodies, have a limited use in cancer therapy because of their large size and limited ability to penetrate the tumor tissue.

**Figure 9:**
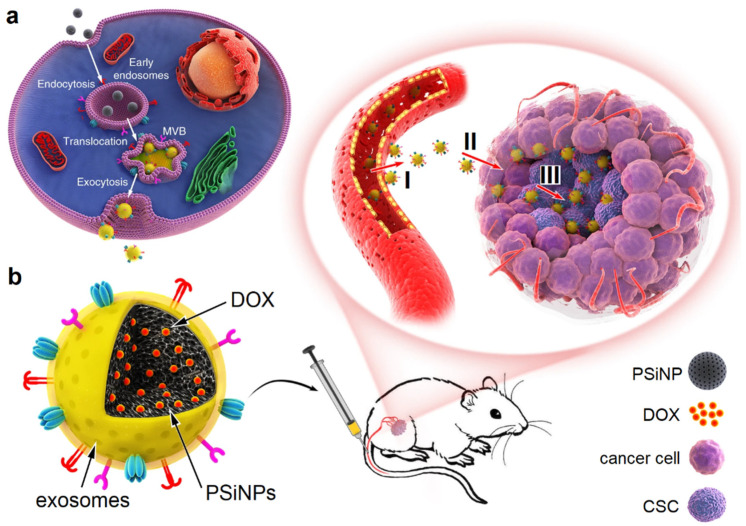
Schematic illustration of exosome-sheathed porous silicon nanoparticles (PSiNPs) as nano-carriers in targeted tumor drug delivery: (**a**) DOX-PSiNPs are endocytosed into cancer cells, localized in multivesicular bodies (MVBs), and formed into autophagosomes. After fusion with the cell membrane, the exosome-sheathed DOX-PSiNPs are exocytosed to the extracellular space for uptake by other cells in the tumor tissue; (**b**) schematic illustration of the intravenous injection of exosome-sheathed DOX-PSiNPs in the tail vein of the tumor-bearing mouse, showing an efficient tumor accumulation (I), tumor penetration (II), and cell internalization (III) of exosome-sheathed DOX-PSiNPs. Adapted from [125] under the terms of the Creative Commons CC BY license.

Exosomes can be engineered to target CSCs in cancer therapy. Autophagy is an intracellular process for the clearance, degradation, exocytosis of damaged organelles and cell components, or the ejection of foreign bodies [124]. In one study, luminescent porous silicon NPs (PsiNPs) with an average diameter of 150 nm, generated by electrochemical etching, were loaded with DOX [125]. Next, the DOX-PSiNPs were incubated with human hepatocarcinoma cells to undergo endocytosis (Figure 9). The experimental results showed that the endocytosed PSiNPs were localized to multivesicular bodies (MVBs) and induced the formation of autophagosomes, which led to autophagy. Following washing and incubating the cells with a fresh medium, the endocytosed DOX-PSiNPs fused with the cell membrane and exocytosed to the extracellular space as exosome-sheathed DOX-PSiNPs. The treatment of subcutaneous, orthotopic or metastatic tumors with exosome-sheathed DOX-PSiNPs resulted in an enhanced tumor penetration, cellular uptake, DOX accumulation in CSCs, and elimination of CSCs [125].

It has been reported that exosomes carry and translocate membrane proteins from tumor cells in one organ to healthy cells in other organs to serve as seed surface receptors for the landing and proliferation of migrated cancer cells [126]. The membrane protein, p120-Catenin (p120ctn), is downregulated in exosomes exocytosed by hepatocellular carcinoma cells (HCCs), as compared to exosomes exocytosed by healthy liver cells [127], implying that the downregulation of p120ctn simulates tumor growth and progression. HCCs treated with exosomes isolated from hepatoma cells transfected with p120ctn-overexpressing lentivirus formed fewer colonies and inhibited the proliferation and migration of HCCs. Further, the p120-ctn overexpressing exosomes reduced tumor growth in a hepatocarcinoma xenograft mouse model [127]. The expression of miR-21-5p was upregulated in the EVs isolated from M2-polarized tumor-associated macrophages [128]. The miR-21-5p upregulated exosomes derived from M2 polarized macrophages enhanced the proliferation and activity of pancreatic CSCs [129]. Further, the downregulation of miR-21-5p in EVs isolated from M2 polarized macrophages inhibited colony formation by pancreatic CSCs in vitro and tumor growth in vivo in an exosome-incubated pancreatic CSC xenograft mouse model [129].

The development of novel targeted therapies that reverse drug resistance in CSCs is highly beneficial for cancer patients. Melanoma-derived CSCs treated with cisplatin-incubated extracellular drug-packaging microparticles, isolated from non-small cell lung cancer cells, reversed drug resistance. This effect was attributed to a downregulation of drug efflux and increased nuclear uptake [130]. While exosomes and other EVs have an enormous potential in targeted CSC therapy, more research needs to be conducted to identify and remove those biomolecules carried by EVs that stimulate tumor growth and metastasis.

## 8. Conclusions

NPs are very attractive as a carrier for targeting drugs to cancer tissue through the leaky tumor vasculature (EPR effect). The surface modification of NPs with water-soluble polymers, such as PEG, PAA, and DEX, has been used to evade the uptake of NPs by the MPS system, increase the residence time in circulation, and increase their uptake through the vasculature. Aside from surface modification, the drug-loaded NPs are targeted to CSCs within the tumor tissue by conjugation with antibodies or ligands against biomarkers, surface receptors, enzymes, and proteins associated with CSC signaling pathways. As most CSC signaling pathways and associated biomarkers are shared with normal stem cells, dual-targeting using two ligands/antibodies against those biomarkers significantly enhances CSC uptake while reducing off-target toxicity toward normal stem cells. Polymeric NPs based on PLA, PLGA, PEG, their copolymers, polylysine, lipids, hyaluronic acid, and liposomes have successfully been used as carriers for targeting therapeutic agents to CSCs. In contrast to polymeric NPs that have a broad size distribution, the size distribution of inorganic NPs tends to be narrow, which improves their transport within the tumor tissue for targeting and uptake by CSCs. Inorganic NPs based on gold, iron oxide, and silica have been used as carriers for drug targeting to CSCs, as well as imaging. Multifunctional protein NPs, due to their degradability by natural enzymes, tunable self-assembly, and natural ability to penetrate the cell membrane, are attractive in connection with the delivery of amino-acid-based therapeutic agents, such as ribosome-inactivating proteins (RIP), to inhibit protein synthesis and cell growth in cancer cells. ADCs are highly effective in eliminating metastatic and recurrent cancers with the selection of antibodies with a high specificity against CSC surface receptors, an appropriate choice of therapeutic agents, and the proper selection of enzymatically degradable linkers for intracellular drug delivery to CSCs. Exosomes and other EVs, due to their low immunogenicity, long circulation time, and high loading capacity, are very attractive as a carrier for the delivery of functional proteins, mRNAs, miRNAs, and small DNA fragments to CSCs for reversing tumor progression, because EVs facilitate cell–cell communication by acting as antigen-presenting vesicles. Drug-loaded polymeric, inorganic or protein NPs, ADCs, and EVs that selectively interact with multiple surface receptors’ tumor-associated stem cells provide the prospect of an enhanced drug bioavailability and uptake in tumor tissue, with fewer undesired side effects in healthy tissue, thus improving the quality of life of cancer patients.

## Figures and Tables

**Table 1:** Target antigens for ADCs under development or in the clinic for cancer therapy [106].

Indication	Targets
Acute myeloid leukaemia	CD25, CD33, CD123 (IL-3Rα), FLT3
Breast cancer	CD25, CD174, CD197 (CCR7), CD205 (Ly75), CD228 (P79, SEMF), c-MET, CRIPTO, ErbB2 (HER2), ErbB3 (HER3), FLOR1 (FRα), Globo H, GPNMB, IGF-1R, integrin β-6, PTK7 (CCK4), nectin-4 (PVRL4), ROR2, SLC39A6 (LIV1A ZIP6)
Bladder cancer	CD25, CD205(Ly75)
Colorectal cancer	CD74, CD174, CD166, CD227 (MUC-1), CD326 (Epcam), CEACAM5, CRIPTO, FAP, ED-B, ErbB3 (HER3)
Gastric cancer	CD25, CD197 (CCR7), CD228 (P79, SEMF), FLOR1(FRα), Globo H, GRP20, GCC, SLC39A6 (LIV1A ZIP6)
Gliomas GIII and GIV	CD25, EGFR
Head and neck cancer	CD71 (transferrin R), CD197 (CCR7), EGFR, SLC39A6 (LIV1A ZIP6)
Hodgkin’s lymphoma	CD25, CD30, CD197 (CCR7)
Lung cancer	Axl, alpha v beta6, CD25, CD56, CD71 (transferrin R), CD228 (P79, SEMF), CD326, CRIPTO, EGFR, ErbB3 (HER3), FAP, Globo H, GD2, IGF-1R, integrin β-6, mesothelin, PTK7 (CCK4), ROR2, SLC34A2 (NaPi2b), SLC39A6 (LIV1A ZIP6)
Liver cancer	CD276 (B7-H3), c-MET
Melanoma	CD276 (B7-H3), GD2, GPNMB, ED-B, PMEL 17, endothelin B receptor
Mesothelioma	Mesothelin, CD228 (P79, SEMF)
Multiple Myeloma	CD38, CD46 (MCP), CD56, CD74, CD138, CD269 (BCMA), endothelin B receptor
Non-Hodgkin Lymphoma	CD19, CD20, CD22, CD25, CD30, CD37, CD70, CD71 (transferrin R), CD72, CD79, CD180, CD205 (Ly75), ROR1
Ovarian cancer	CA125(MUC16), CD142 (TF), CD205 (Ly75), FLOR1(FRα), Globo H, mesothelin, PTK7 (CCK4)
Pancreatic cancer	CD25, CD71 (transferrin R), CD74, CD227 (MUC1), CD228 (P79, SEMF), GRP20, GCC, IGF-1R, integrin β-6, nectin-4 (PVRL4), SLC34A2 (NaPi2b), SLC44A4, alpha v beta6, mesothelin
Prostate cancer	CD46 (MCP), PSMA, STEAP-1, SLC44A4, TENB2
Renal cancer	AGS-16, EGFR, c-MET, CAIX, CD70, FLOR1 (FRα)

## References

[1] Duan H., Liu Y., Gao Z., Huang W. (2021). Recent advances in drug delivery systems for targeting cancer stem cells. Acta Pharm. Sin. B.

[2] World Health Organization (2020). WHO Report on Cancer: Setting Priorities, Investing Wisely and Providing Care for All.

[3] Asghari F., Khademi R., Ranjbar F.E., Malekshahi Z.V., Majidi R.F. (2019). Application of Nanotechnology in Targeting of Cancer Stem Cells: A Review. Int. J. Stem Cells.

[4] Al-Hajj M., Wicha M.S., Benito-Hernandez A., Morrison S.J., Clarke M.F. (2003). Prospective identification of tumorigenic breast cancer cells. Proc. Natl. Acad. Sci. USA.

[5] O’Brien C.A., Pollett A., Gallinger S., Dick J.E. (2007). A human colon cancer cell capable of initiating tumour growth in immunodeficient mice. Nature.

[6] Kim C.F.B., Jackson E.L., Woolfenden A.E., Lawrence S., Babar I., Vogel S., Crowley D., Bronson R.T., Jacks T. (2005). Identification of bronchioalveolar stem cells in normal lung and lung cancer. Cell.

[7] Collins A.T., Maitland N.J. (2006). Prostate cancer stem cells. Eur. J. Cancer.

[8] Yang Z.F., Ngai P., Ho D.W., Yu W.C., Ng M.N., Lau C.K., Li M.L., Tam K.H., Lam C.T., Poon R.T. (2008). Identification of local and circulating cancer stem cells in human liver cancer. Hepatology.

[9] Fang D., Nguyen T.K., Leishear K., Finko R., Kulp A.N., Hotz S., Van Belle P.A., Xu X., Elder D.E., Herlyn M. (2005). A tumorigenic subpopulation with stem cell properties in melanomas. Cancer Res..

[10] Singh S.K., Clarke I.D., Terasaki M., Bonn V.E., Hawkins C., Squire J., Dirks P.B. (2003). Identification of a cancer stem cell in human brain tumors. Cancer Res..

[11] Prince M., Sivanandan R., Kaczorowski A., Wolf G., Kaplan M., Dalerba P., Weissman I., Clarke M., Ailles L. (2007). Identification of a subpopulation of cells with cancer stem cell properties in head and neck squamous cell carcinoma. Proc. Natl. Acad. Sci. USA.

[12] Szotek P.P., Pieretti-Vanmarcke R., Masiakos P.T., Dinulescu D.M., Connolly D., Foster R., Dombkowski D., Preffer F., MacLaughlin D.T., Donahoe P.K. (2006). Ovarian cancer side population defines cells with stem cell-like characteristics and Mullerian Inhibiting Substance responsiveness. Proc. Natl. Acad. Sci. USA.

[13] Hermann P.C., Huber S.L., Herrler T., Aicher A., Ellwart J.W., Guba M., Bruns C.J., Heeschen C. (2007). Distinct populations of cancer stem cells determine tumor growth and metastatic activity in human pancreatic cancer. Cell Stem Cell.

[14] Hemmati H.D., Nakano I., Lazareff J.A., Masterman-Smith M., Geschwind D.H., Bronner-Fraser M., Kornblum H.I. (2003). Cancerous stem cells can arise from pediatric brain tumors. Proc. Natl. Acad. Sci. USA.

[15] Zhao C.-Y., Cheng R., Yang Z., Tian Z.-M. (2018). Nanotechnology for cancer therapy based on chemotherapy. Molecules.

[16] Grodzinski P., Kircher M., Goldberg M., Gabizon A. (2019). Integrating nanotechnology into cancer care. ACS Nano.

[17] Li B., Li Q., Mo J., Dai H. (2017). Drug-loaded polymeric nanoparticles for cancer stem cell targeting. Front. Pharmacol..

[18] Hong I.-S., Jang G.-B., Lee H.-Y., Nam J.-S. (2015). Targeting cancer stem cells by using the nanoparticles. Int. J. Nanomed..

[19] Tabassum N., Verma V., Kumar M., Kumar A., Singh B. (2018). Nanomedicine in cancer stem cell therapy: From fringe to forefront. Cell Tissue Res..

[20] Bhartiya D., Patel H., Ganguly R., Shaikh A., Shukla Y., Sharma D., Singh P. (2018). Novel insights into adult and cancer stem cell biology. Stem Cells Dev..

[21] Lau E.Y.-T., Ho N.P.-Y., Lee T.K.-W. (2017). Cancer stem cells and their microenvironment: Biology and therapeutic implications. Stem Cells Int..

[22] Mokhtarzadeh A., Hassanpour S., Vahid Z.F., Hejazi M., Hashemi M., Ranjbari J., Tabarzad M., Noorolyai S., de la Guardia M. (2017). Nano-delivery system targeting to cancer stem cell cluster of differentiation biomarkers. J. Control. Release.

[23] Qin W., Huang G., Chen Z., Zhang Y. (2017). Nanomaterials in targeting cancer stem cells for cancer therapy. Front. Pharmacol..

[24] Soltani F., Ramezani M., Amel Farzad S., Mokhtarzadeh A., Hashemi M. (2017). Comparison study of the effect of alkyl-modified and unmodified PAMAM and PPI dendrimers on solubility and antitumor activity of crocetin. Artif. Cells Nanomed. Biotechnol..

[25] Dalpiaz A., Paganetto G., Botti G., Pavan B. (2020). Cancer stem cells and nanomedicine: New opportunities to combat multidrug resistance?. Drug Discov. Today.

[26] Nunes T., Hamdan D., Leboeuf C., El Bouchtaoui M., Gapihan G., Nguyen T.T., Meles S., Angeli E., Ratajczak P., Lu H. (2018). Targeting cancer stem cells to overcome chemoresistance. Int. J. Mol. Sci..

[27] Dianat-Moghadam H., Heidarifard M., Jahanban-Esfahlan R., Panahi Y., Hamishehkar H., Pouremamali F., Rahbarghazi R., Nouri M. (2018). Cancer stem cells-emanated therapy resistance: Implications for liposomal drug delivery systems. J. Control. Release.

[28] Loureiro R., Mesquita K.A., Magalhães-Novais S., Oliveira P.J., Vega-Naredo I. (2017). Mitochondrial biology in cancer stem cells. Semin. Cancer Biol..

[29] Mokhtarzadeh A., Alibakhshi A., Hashemi M., Hejazi M., Hosseini V., de la Guardia M., Ramezani M. (2017). Biodegradable nano-polymers as delivery vehicles for therapeutic small non-coding ribonucleic acids. J. Control. Release.

[30] Mokhtarzadeh A., Tabarzad M., Ranjbari J., de la Guardia M., Hejazi M., Ramezani M. (2016). Aptamers as smart ligands for nano-carriers targeting. TrAC Trends Anal. Chem..

[31] Mokhtarzadeh A., Alibakhshi A., Yaghoobi H., Hashemi M., Hejazi M., Ramezani M. (2016). Recent advances on biocompatible and biodegradable nanoparticles as gene carriers. Expert Opin. Biol. Ther..

[32] Iyer A.K., Singh A., Ganta S., Amiji M.M. (2013). Role of integrated cancer nanomedicine in overcoming drug resistance. Adv. Drug Deliv. Rev..

[33] Pavan B., Paganetto G., Rossi D., Dalpiaz A. (2014). Multidrug resistance in cancer or inefficacy of neuroactive agents: Innovative strategies to inhibit or circumvent the active efflux transporters selectively. Drug Discov. Today.

[34] Yang Z., Sun N., Cheng R., Zhao C., Liu J., Tian Z. (2017). Hybrid nanoparticles coated with hyaluronic acid lipoid for targeted co-delivery of paclitaxel and curcumin to synergistically eliminate breast cancer stem cells. J. Mater. Chem. B.

[35] Li J., Xu W., Yuan X., Chen H., Song H., Wang B., Han J. (2017). Polymer–lipid hybrid anti-HER2 nanoparticles for targeted salinomycin delivery to HER2-positive breast cancer stem cells and cancer cells. Int. J. Nanomed..

[36] Abou-ElNaga A., Mutawa G., El-Sherbiny I.M., Abd-ElGhaffar H., Allam A.A., Ajarem J., Mousa S.A. (2017). Novel nano-therapeutic approach actively targets human ovarian cancer stem cells after xenograft into nude mice. Int. J. Mol. Sci..

[37] Verma R.K., Yu W., Singh S.P., Shankar S., Srivastava R.K. (2015). Anthothecol-encapsulated PLGA nanoparticles inhibit pancreatic cancer stem cell growth by modulating sonic hedgehog pathway. Nanomed. Nanotechnol. Biol. Med..

[38] Ni M., Xiong M., Zhang X., Cai G., Chen H., Zeng Q., Yu Z. (2015). Poly (lactic-co-glycolic acid) nanoparticles conjugated with CD133 aptamers for targeted salinomycin delivery to CD133+ osteosarcoma cancer stem cells. Int. J. Nanomed..

[39] Muntimadugu E., Kumar R., Saladi S., Rafeeqi T.A., Khan W. (2016). CD44 targeted chemotherapy for co-eradication of breast cancer stem cells and cancer cells using polymeric nanoparticles of salinomycin and paclitaxel. Colloids Surf. B Biointerfaces.

[40] Sun N., Zhao C., Cheng R., Liu Z., Li X., Lu A., Tian Z., Yang Z. (2018). Cargo-free nanomedicine with pH sensitivity for codelivery of DOX conjugated prodrug with SN38 to synergistically eradicate breast cancer stem cells. Mol. Pharm..

[41] Wang M., Xie F., Wen X., Chen H., Zhang H., Liu J., Zhang H., Zou H., Yu Y., Chen Y. (2017). Therapeutic PEG-ceramide nanomicelles synergize with salinomycin to target both liver cancer cells and cancer stem cells. Nanomedicine.

[42] Ke X.-Y., Ng V.W.L., Gao S.-J., Tong Y.W., Hedrick J.L., Yang Y.Y. (2014). Co-delivery of thioridazine and doxorubicin using polymeric micelles for targeting both cancer cells and cancer stem cells. Biomaterials.

[43] Mi Y., Huang Y., Deng J. (2018). The enhanced delivery of salinomycin to CD133+ ovarian cancer stem cells through CD133 antibody conjugation with poly (lactic-co-glycolic acid)-poly (ethylene glycol) nanoparticles. Oncol. Lett..

[44] Chen D., Pan X., Xie F., Lu Y., Zou H., Yin C., Zhang Y., Gao J. (2018). Codelivery of doxorubicin and elacridar to target both liver cancer cells and stem cells by polylactide-co-glycolide/d-alpha-tocopherol polyethylene glycol 1000 succinate nanoparticles. Int. J. Nanomed..

[45] Zhang Y., Zhang Q., Sun J., Liu H., Li Q. (2017). The combination therapy of salinomycin and gefitinib using poly (d, l-lactic-co-glycolic acid)-poly (ethylene glycol) nanoparticles for targeting both lung cancer stem cells and cancer cells. OncoTargets Ther..

[46] Qiao S., Zhao Y., Geng S., Li Y., Hou X., Liu Y., Lin F.-H., Yao L., Tian W. (2016). A novel double-targeted nondrug delivery system for targeting cancer stem cells. Int. J. Nanomed..

[47] Chen H., Lin J., Shan Y., Zhengmao L. (2019). The promotion of nanoparticle delivery to two populations of gastric cancer stem cells by CD133 and CD44 antibodies. Biomed. Pharmacother..

[48] Xu H.-L., Fan Z.-L., ZhuGe D.-L., Tong M.-Q., Shen B.-X., Lin M.-T., Zhu Q.-Y., Jin B.-H., Sohawon Y., Yao Q. (2018). Ratiometric delivery of two therapeutic candidates with inherently dissimilar physicochemical property through pH-sensitive core–shell nanoparticles targeting the heterogeneous tumor cells of glioma. Drug Deliv..

[49] Jang E., Kim E., Son H.-Y., Lim E.-K., Lee H., Choi Y., Park K., Han S., Suh J.-S., Huh Y.-M. (2016). Nanovesicle-mediated systemic delivery of microRNA-34a for CD44 overexpressing gastric cancer stem cell therapy. Biomaterials.

[50] Sun R., Shen S., Zhang Y.-J., Xu C.-F., Cao Z.-T., Wen L.-P., Wang J. (2016). Nanoparticle-facilitated autophagy inhibition promotes the efficacy of chemotherapeutics against breast cancer stem cells. Biomaterials.

[51] Sun R., Liu Y., Li S.-Y., Shen S., Du X.-J., Xu C.-F., Cao Z.-T., Bao Y., Zhu Y.-H., Li Y.-P. (2015). Co-delivery of all-trans-retinoic acid and doxorubicin for cancer therapy with synergistic inhibition of cancer stem cells. Biomaterials.

[52] Li S.-Y., Sun R., Wang H.-X., Shen S., Liu Y., Du X.-J., Zhu Y.-H., Jun W. (2015). Combination therapy with epigenetic-targeted and chemotherapeutic drugs delivered by nanoparticles to enhance the chemotherapy response and overcome resistance by breast cancer stem cells. J. Control. Release.

[53] Mukherjee A., Waters A.K., Kalyan P., Achrol A.S., Kesari S., Yenugonda V.M. (2019). Lipid–polymer hybrid nanoparticles as a next-generation drug delivery platform: State of the art, emerging technologies, and perspectives. Int. J. Nanomed..

[54] Zeng Y.-B., Yu Z.-C., He Y.-N., Zhang T., Du L.-B., Dong Y.-M., Chen H.-W., Zhang Y.-Y., Wang W.-Q. (2018). Salinomycin-loaded lipid-polymer nanoparticles with anti-CD20 aptamers selectively suppress human CD20+ melanoma stem cells. Acta Pharmacol. Sin..

[55] Chen F., Zeng Y., Qi X., Chen Y., Ge Z., Jiang Z., Zhang X., Dong Y., Chen H., Yu Z. (2018). Targeted salinomycin delivery with EGFR and CD133 aptamers based dual-ligand lipid-polymer nanoparticles to both osteosarcoma cells and cancer stem cells. Nanomed. Nanotechnol. Biol. Med..

[56] Yu L.-Y., Shen Y.-A., Chen M.-H., Wen Y.-H., Hsieh P.-I., Lo C.-L. (2019). The feasibility of ROS-and GSH-responsive micelles for treating tumor-initiating and metastatic cancer stem cells. J. Mater. Chem. B.

[57] Yang R., Mondal G., Wen D., Mahato R.I. (2017). Combination therapy of paclitaxel and cyclopamine polymer-drug conjugates to treat advanced prostate cancer. Nanomed. Nanotechnol. Biol. Med..

[58] Ning S.-T., Lee S.-Y., Wei M.-F., Peng C.-L., Lin S.Y.-F., Tsai M.-H., Lee P.-C., Shih Y.-H., Lin C.-Y., Luo T.-Y. (2016). Targeting colorectal cancer stem-like cells with anti-CD133 antibody-conjugated SN-38 nanoparticles. ACS Appl. Mater. Interfaces.

[59] Debele T.A., Yu L.-Y., Yang C.-S., Shen Y.-A., Lo C.-L. (2018). pH-and GSH-sensitive hyaluronic acid-MP conjugate micelles for intracellular delivery of doxorubicin to colon cancer cells and cancer stem cells. Biomacromolecules.

[60] Gaio E., Conte C., Esposito D., Reddi E., Quaglia F., Moret F. (2020). CD44 Targeting Mediated by Polymeric Nanoparticles and Combination of Chlorine TPCS2a-PDT and Docetaxel-Chemotherapy for Efficient Killing of Breast Differentiated and Stem Cancer Cells In Vitro. Cancers.

[61] Gao J., Liu J., Xie F., Lu Y., Yin C., Shen X. (2019). Co-Delivery of Docetaxel and Salinomycin to Target Both Breast Cancer Cells and Stem Cells by PLGA/TPGS Nanoparticles. Int. J. Nanomed..

[62] Wang Z., Sun M., Li W., Fan L., Zhou Y., Hu Z. (2020). A novel CD133-and EpCAM-targeted liposome with redox-responsive properties capable of synergistically eliminating liver cancer stem cells. Front. Chem..

[63] Sun X., Chen Y., Zhao H., Qiao G., Liu M., Zhang C., Cui D., Ma L. (2018). Dual-modified cationic liposomes loaded with paclitaxel and survivin siRNA for targeted imaging and therapy of cancer stem cells in brain glioma. Drug Deliv..

[64] Yang R., Lu M., Ming L., Chen Y., Cheng K., Zhou J., Jiang S., Lin Z., Chen D. (2020). 89Zr-Labeled Multifunctional Liposomes Conjugate Chitosan for PET-Trackable Triple-Negative Breast Cancer Stem Cell Targeted Therapy. Int. J. Nanomed..

[65] Yang Z., Sun N., Cheng R., Zhao C., Liu Z., Li X., Liu J., Tian Z. (2017). pH multistage responsive micellar system with charge-switch and PEG layer detachment for co-delivery of paclitaxel and curcumin to synergistically eliminate breast cancer stem cells. Biomaterials.

[66] Wang H., Agarwal P., Zhao S., Xu R.X., Yu J., Lu X., He X. (2015). Hyaluronic acid-decorated dual responsive nanoparticles of Pluronic F127, PLGA, and chitosan for targeted co-delivery of doxorubicin and irinotecan to eliminate cancer stem-like cells. Biomaterials.

[67] Li Y., Shi S., Ming Y., Wang L., Li C., Luo M., Li Z., Li B., Chen J. (2018). Specific cancer stem cell-therapy by albumin nanoparticles functionalized with CD44-mediated targeting. J. Nanobiotechnol..

[68] Usacheva M., Swaminathan S.K., Kirtane A.R., Panyam J. (2014). Enhanced photodynamic therapy and effective elimination of cancer stem cells using surfactant–polymer nanoparticles. Mol. Pharm..

[69] Rao W., Wang H., Han J., Zhao S., Dumbleton J., Agarwal P., Zhang W., Zhao G., Yu J., Zynger D.L. (2015). Chitosan-decorated doxorubicin-encapsulated nanoparticle targets and eliminates tumor reinitiating cancer stem-like cells. ACS Nano.

[70] Wu P., Liu Q., Wang Q., Qian H., Yu L., Liu B., Li R. (2018). Novel silk fibroin nanoparticles incorporated silk fibroin hydrogel for inhibition of cancer stem cells and tumor growth. Int. J. Nanomed..

[71] Zhao P., Dong S., Bhattacharyya J., Chen M. (2014). iTEP nanoparticle-delivered salinomycin displays an enhanced toxicity to cancer stem cells in orthotopic breast tumors. Mol. Pharm..

[72] Mitchell M.J., Billingsley M.M., Haley R.M., Wechsler M.E., Peppas N.A., Langer R. (2021). Engineering precision nanoparticles for drug delivery. Nat. Rev. Drug. Discov..

[73] Sharma A., Goyal A.K., Rath G. (2018). Recent advances in metal nanoparticles in cancer therapy. J. Drug Target..

[74] McNeil S.E. (2016). Evaluation of nanomedicines: Stick to the basics. Nat. Rev. Mater..

[75] Mohd-Zahid M.H., Mohamud R., Abdullah C.A.C., Lim J., Alem H., Hanaffi W.N.W., Iskandar Z. (2020). Colorectal cancer stem cells: A review of targeted drug delivery by gold nanoparticles. RSC Adv..

[76] Murphy C.J., Gole A.M., Stone J.W., Sisco P.N., Alkilany A.M., Goldsmith E.C., Baxter S.C. (2008). Gold nanoparticles in biology: Beyond toxicity to cellular imaging. Acc. Chem. Res..

[77] Safwat M.A., Soliman G.M., Sayed D., Attia M.A. (2016). Gold nanoparticles enhance 5-fluorouracil anticancer efficacy against colorectal cancer cells. Int. J. Pharm..

[78] Zhao Y., Zhao W., Lim Y.C., Liu T. (2019). Salinomycin-loaded gold nanoparticles for treating cancer stem cells by ferroptosis-induced cell death. Mol. Pharm..

[79] Hu C., Niestroj M., Yuan D., Chang S., Chen J. (2015). Treating cancer stem cells and cancer metastasis using glucose-coated gold nanoparticles. Int. J. Nanomed..

[80] Yi Y., Kim H.J., Zheng M., Mi P., Naito M., Kim B.S., Min H.S., Hayashi K., Perche F., Toh K. (2019). Glucose-linked sub-50-nm unimer polyion complex-assembled gold nanoparticles for targeted siRNA delivery to glucose transporter 1-overexpressing breast cancer stem-like cells. J. Control. Release.

[81] Kim H.J., Takemoto H., Yi Y., Zheng M., Maeda Y., Chaya H., Hayashi K., Mi P., Pittella F., Christie R.J. (2014). Precise Engineering of siRNA Delivery Vehicles to Tumors Using Polyion Complexes and Gold Nanoparticles. ACS Nano.

[82] Yi Y., Kim H.J., Mi P., Zheng M., Takemoto H., Toh K., Kim B.S., Hayashi K., Naito M., Matsumoto Y. (2016). Targeted systemic delivery of siRNA to cervical cancer model using cyclic RGD-installed unimer polyion complex-assembled gold nanoparticles. J. Control. Release.

[83] Patra C.R., Bhattacharya R., Mukherjee P. (2009). Fabrication and functional characterization of goldnanoconjugates for potential application in ovarian cancer. J. Mater. Chem..

[84] Chanda N., Kattumuri V., Shukla R., Zambre A., Katti K., Upendran A., Kulkarni R.R., Kan P., Fent G.M., Casteel S.W. (2010). Bombesin functionalized gold nanoparticles show in vitro and in vivo cancer receptor specificity. Proc. Natl. Acad. Sci. USA.

[85] Li J.-L., Wang L., Liu X.-Y., Zhang Z.-P., Guo H.-C., Liu W.-M., Tang S.-H. (2009). In vitro cancer cell imaging and therapy using transferrin-conjugated gold nanoparticles. Cancer Lett..

[86] Latorre A., Latorre A., Castellanos M., Rodriguez Diaz C., Lazaro-Carrillo A., Aguado T., Lecea M., Romero-Pérez S., Calero M., Sanchez-Puelles J.M. (2019). Multifunctional albumin-stabilized gold nanoclusters for the reduction of cancer stem cells. Cancers.

[87] El-Boubbou K. (2018). Magnetic iron oxide nanoparticles as drug carriers: Preparation, conjugation and delivery. Nanomedicine.

[88] Su Z., Liu D., Chen L., Zhang J., Ru L., Chen Z., Gao Z., Wang X. (2019). CD44-targeted magnetic nanoparticles kill head and neck squamous cell carcinoma stem cells in an alternating magnetic field. Int. J. Nanomed..

[89] Miller-Kleinhenz J., Guo X., Qian W., Zhou H., Bozeman E.N., Zhu L., Ji X., Wang Y.A., Styblo T., O’Regan R. (2018). Dual-targeting Wnt and uPA receptors using peptide conjugated ultra-small nanoparticle drug carriers inhibited cancer stem-cell phenotype in chemo-resistant breast cancer. Biomaterials.

[90] Chen L., Liu M., Zhou Q., Li X. (2020). Recent developments of mesoporous silica nanoparticles in biomedicine. Emergent Mater..

[91] Zhou Y., Quan G., Wu Q., Zhang X., Niu B., Wu B., Huang Y., Pan X., Wu C. (2018). Mesoporous silica nanoparticles for drug and gene delivery. Acta Pharm. Sin. B.

[92] Pan Y., Zhou S., Li Y., Parshad B., Li W., Haag R. (2021). Novel dendritic polyglycerol-conjugated, mesoporous silica-based targeting nanocarriers for co-delivery of doxorubicin and tariquidar to overcome multidrug resistance in breast cancer stem cells. J. Control. Release.

[93] Tsai P.-H., Wang M.-L., Chang J.-H., Yarmishyn A.A., Nhi Nguyen P.N., Chen W., Chien Y., Huo T.-I., Mou C.-Y., Chiou S.-H. (2019). Dual delivery of HNF4α and cisplatin by mesoporous silica nanoparticles inhibits cancer pluripotency and tumorigenicity in hepatoma-derived CD133-expressing stem cells. ACS Appl. Mater. Interfaces.

[94] Shen J.L., Wolfram J., Ferrari M., Shen H.F. (2017). Taking the vehicle out of drug delivery. Mater. Today.

[95] Volta-Duran E., Serna N., Sanchez-Garcia L., Avino A., Sanchez J.M., Lopez-Laguna H., Cano-Garrido O., Casanova I., Mangues R., Eritja R. (2021). Design and engineering of tumor-targeted, dual-acting cytotoxic nanoparticles. Acta Biomater..

[96] Casanova S., Unzueta U., Arroyo-Solera I., Cespedes M.V., Villaverde A., Mangues R., Vazquez E. (2019). Protein-driven nanomedicines in oncotherapy. Curr. Opin. Pharmacol..

[97] Deci M.B., Liu M.X., Gonya J., Lee C.J., Li T.Y., Ferguson S.W., Bonacquisti E.E., Wang J.L., Nguyen J. (2019). Carrier-Free CXCR4-Targeted Nanoplexes Designed for Polarizing Macrophages to Suppress Tumor Growth. Cell Mol. Bioeng..

[98] Volta-Duran E., Cano-Garrido O., Serna N., Lopez-Laguna H., Sanchez-Garcia L., Pesarrodona M., Sanchez-Chardi A., Mangues R., Villaverde A., Vazquez E. (2020). Controlling self-assembling and tumor cell-targeting of protein-only nanoparticles through modular protein engineering. Sci. China Mater..

[99] Shipunova V.O., Kotelnikova P.A., Aghayeva U.F., Stremovskiy O.A., Novikov I.A., Schulga A.A., Nikitin M.P., Deyev S.M. (2019). Self-assembling nanoparticles biofunctionalized with magnetite-binding protein for the targeted delivery to HER2/neu overexpressing cancer cells. J. Magn. Mater..

[100] Liu Q., Zhang L., Ji X.R., Shin M.C., Xie S.P., Pan B.Y., Yu F., Zhao J.W., Yang V.C. (2020). A self-assembly and stimuli-responsive fusion gelonin delivery system for tumor treatment. J. Ind. Eng. Chem..

[101] Serna N., Sanchez-Garcia L., Unzueta U., Diaz R., Vazquez E., Mangues R., Villaverde A. (2018). Protein-Based Therapeutic Killing for Cancer Therapies. Trends Biotechnol..

[102] Wang Z.C., Zhi K.K., Ding Z.Y., Sun Y., Li S., Li M.Y., Pu K.F., Zou J. (2021). Emergence in protein derived nanomedicine as anticancer therapeutics: More than a tour de force. Semin. Cancer Biol..

[103] Villela-Martinez L.M., Velez-Ayala A.K., Lopez-Sanchez R.D., Martinez-Cardona J.A., Hernandez-Hernandez J.A. (2017). Advantages of Drug Selective Distribution in Cancer Treatment: Brentuximab Vedotin. Int J. Pharmacol..

[104] Walko C.M., West H. (2019). Antibody Drug Conjugates for Cancer Treatment. Jama Oncol..

[105] Fernandes E., Ferreira J.A., Peixoto A., Lima L., Barroso S., Sarmento B., Santos L.L. (2015). New trends in guided nanotherapies for digestive cancers: A systematic review. J. Control. Release.

[106] Hafeez U., Parakh S., Gan H.K., Scott A.M. (2020). Antibody-Drug Conjugates for Cancer Therapy. Molecules.

[107] Chari R.V.J., Miller M.L., Widdison W.C. (2014). Antibody-Drug Conjugates: An Emerging Concept in Cancer Therapy. Angew. Chem. Int. Ed..

[108] Agarwal P., Bertozzi C.R. (2015). Site-Specific Antibody-Drug Conjugates: The Nexus of Biciorthogonal Chemistry, Protein Engineering, and Drug Development. Bioconjugate Chem..

[109] Narayanaswamy R., Torchilin V.P. (2021). Targeted Delivery of Combination Therapeutics Using Monoclonal Antibody 2C5-Modified Immunoliposomes for Cancer Therapy. Pharm Res. Dordr..

[110] Masoumi E., Jafarzadeh L., Mirzaei H.R., Alishah K., Fallah-Mehrjardi K., Rostamian H., Khakpoor-Koosheh M., Meshkani R., Noorbakhsh F., Hadjati J. (2020). Genetic and pharmacological targeting of A2a receptor improves function of anti-mesothelin CAR T cells. J. Exp. Clin. Cancer Res..

[111] Golfier S., Kopitz C., Kahnert A., Heisler I., Schatz C.A., Stelte-Ludwig B., Mayer-Bartschmid A., Unterschemmann K., Bruder S., Linden L. (2014). Anetumab Ravtansine: A Novel Mesothelin-Targeting Antibody-Drug Conjugate Cures Tumors with Heterogeneous Target Expression Favored by Bystander Effect. Mol. Cancer Ther..

[112] Ogitani Y., Aida T., Hagihara K., Yamaguchi J., Ishii C., Harada N., Soma M., Okamoto H., Oitate M., Arakawa S. (2016). DS-8201a, A Novel HER2-Targeting ADC with a Novel DNA Topoisomerase I Inhibitor, Demonstrates a Promising Antitumor Efficacy with Differentiation from T-DM1. Clin. Cancer Res..

[113] Millar R., Kilbey A., Remak S.J., Severson T.M., Dhayade S., Sandilands E., Foster K., Bryant D.M., Blyth K., Coffelt S.B. (2020). The MSP-RON axis stimulates cancer cell growth in models of triple negative breast cancer. Mol. Oncol..

[114] Tong X.M., Feng L., Suthe S.R., Weng T.H., Hu C.Y., Liu Y.Z., Wu Z.G., Wang M.H., Yao H.P. (2019). Therapeutic efficacy of a novel humanized antibody-drug conjugate recognizing plexin-semaphorin-integrin domain in the RON receptor for targeted cancer therapy. J. Immunother. Cancer.

[115] Godwin C.D., Laszlo G.S., Wood B.L., Correnti C.E., Bates O.M., Garling E.E., Mao Z.J., Beddoe M.E., Lunn M.C., Humbert O. (2020). The CD33 splice isoform lacking exon 2 as therapeutic target in human acute myeloid leukemia. Leukemia.

[116] Kovtun Y., Noordhuis P., Whiteman K.R., Watkins K., Jones G.E., Harvey L., Lai K.C., Portwood S., Adams S., Sloss C.M. (2018). IMGN779, a Novel CD33-Targeting Antibody-Drug Conjugate with DNA-Alkylating Activity, Exhibits Potent Antitumor Activity in Models of AML. Mol. Cancer Ther..

[117] Gupta R., Leon F., Rauth S., Batra S.K., Ponnusamy M.P. (2020). A Systematic Review on the Implications of O-linked Glycan Branching and Truncating Enzymes on Cancer Progression and Metastasis. Cells.

[118] Starbuck K., Al-Alem L., Eavarone D.A., Hernandez S.F., Bellio C., Prendergast J.M., Stein J., Dransfield D.T., Zarrella B., Growdon W.B. (2018). Treatment of ovarian cancer by targeting the tumor stem cell-associated carbohydrate antigen, Sialyl-Thomsen-nouveau. Oncotarget.

[119] Hernandez-Oller L., Seras-Franzoso J., Andrade F., Rafael D., Abasolo I., Gener P., Schwartz S. (2020). Extracellular Vesicles as Drug Delivery Systems in Cancer. Pharmaceutics.

[120] Xiao Y.W., Zheng L., Zou X.F., Wang J.G., Zhong J.N., Zhong T.Y. (2019). Extracellular vesicles in type 2 diabetes mellitus: Key roles in pathogenesis, complications, and therapy. J. Extracell Vesicles.

[121] Gener P., Callejo P.G., Seras-Franzoso J., Andrade F., Rafael D., Abasolo I., Schwartz S.J. (2020). The potential of nanomedicine to alter cancer stem cell dynamics: The impact of extracellular vesicles. Nanomedicine.

[122] Wang J.H., Zheng Y.J., Zhao M. (2017). Exosome-Based Cancer Therapy: Implication for Targeting Cancer Stem Cells. Front. Pharmacol..

[123] Smyth T.J., Redzic J.S., Michael W.B., Anchordoquy T.J. (2014). Examination of the specificity of tumor cell derived exosomes with tumor cells in vitro. Biochim. Biophys. Acta Biomembr..

[124] Morishita H., Mizushima N. (2019). Diverse Cellular Roles of Autophagy. Annu Rev. Cell Dev. Biol.

[125] Yong T.Y., Zhang X.Q., Bie N.N., Zhang H.B., Zhang X.T., Li F.Y., Hakeem A., Hu J., Gan L., Santos H.A. (2019). Tumor exosome-based nanoparticles are efficient drug carriers for chemotherapy. Nat. Commun..

[126] Zhang H.Y., Deng T., Liu R., Bai M., Zhou L.K., Wang X., Li S., Wang X.Y., Yang H., Li J.L. (2017). Exosome-delivered EGFR regulates liver microenvironment to promote gastric cancer liver metastasis. Nat. Commun..

[127] Cheng Z.J., Lei Z.Q., Yang P.H., Si A.F., Xiang D.M., Tang X.W., Guo G.M., Zhou J.H., Huser N. (2019). Exosome-transmitted p120-catenin suppresses hepatocellular carcinoma progression via STAT3 pathways. Mol. Carcinog..

[128] Lin F., Yin H.B., Li X.Y., Zhu G.M., He W.Y., Gou X. (2019). Bladder cancer cellsecreted exosomal miR21 activates the PI3K/AKT pathway in macrophages to promote cancer progression. Int J. Oncol..

[129] Chang J., Li H.J., Zhu Z.C., Mei P., Hu W.M., Xiong X.C., Tao J. (2021). microRNA-21-5p from M2 macrophage-derived extracellular vesicles promotes the differentiation and activity of pancreatic cancer stem cells by mediating KLF3. Cell Biol. Toxicol..

[130] Ma J.W., Zhang Y., Tang K., Zhang H.F., Yin X.N., Li Y., Xu P.W., Sun Y.L., Ma R.H., Ji T.T. (2016). Reversing drug resistance of soft tumor-repopulating cells by tumor cell-derived chemotherapeutic microparticles. Cell Res..

